# Additional Expiratory Resistance Elevates Airway Pressure and Lung Volume during High-Flow Tracheal Oxygen via Tracheostomy

**DOI:** 10.1038/s41598-019-51158-0

**Published:** 2019-10-10

**Authors:** Guang-Qiang Chen, Xiu-Mei Sun, Yu-Mei Wang, Yi-Min Zhou, Jing-Ran Chen, Kun-Ming Cheng, Yan-Lin Yang, Jian-Xin Zhou

**Affiliations:** Department of Critical Care Medicine, Beijing Tiantan Hospital, Capital Medical University, Beijing, China

**Keywords:** Respiration, Respiration, Medical research, Medical research

## Abstract

The standard high-flow tracheal (HFT) interface was modified by adding a 5-cm H_2_O/L/s resistor to the expiratory port. First, in a test lung simulating spontaneous breathing, we found that the modified HFT caused an elevation in airway pressure as a power function of flow. Then, three tracheal oxygen treatments (T-piece oxygen at 10 L/min, HFT and modified HFT at 40 L/min) were delivered in a random crossover fashion to six tracheostomized pigs before and after the induction of lung injury. The modified HFT induced a significantly higher airway pressure compared with that in either T-piece or HFT (*p* < 0.001). Expiratory resistance significantly increased during modified HFT (*p* < 0.05) to a mean value of 4.9 to 6.7 cm H_2_O/L/s. The modified HFT induced significant augmentation in end-expiratory lung volume (*p* < 0.05) and improved oxygenation for lung injury model (*p* = 0.038) compared with the HFT and T-piece. There was no significant difference in esophageal pressure swings, transpulmonary driving pressure or pressure time product among the three treatments (*p* > 0.05). In conclusion, the modified HFT with additional expiratory resistance generated a clinically relevant elevation in airway pressure and lung volume. Although expiratory resistance increased, inspiratory effort, lung stress and work of breathing remained within an acceptable range.

## Introduction

After the discontinuation of mechanical ventilation, approximately 10 to 20% of patients require an artificial airway^1–4^. For patients who cannot protect their own airway, tracheostomy is often performed, and relatively long-term oxygen therapy is required^5,6^. Studies have shown that tracheostomy tubes decrease airway resistance and the work of breathing; however, diminishing the physiological positive end-expiratory pressure (PEEP) via bypassing the larynx and upper airway may also result in a reduction of the functional residual capacity^7,8^. The latter may put the patient at risk for atelectasis and respiratory failure.

High-flow nasal cannula (HFNC) oxygen therapy, which delivers heated and humidified oxygen and air with a maximum flow rate of 60 L/min at a prescribed inspired oxygen concentration, has drawn increasing attention in treating adult patients with mild-to-moderate respiratory failure or after extubation^9–11^. Studies involving bench models^12^, healthy volunteers^13,14^ and surgical patients^15,16^ have demonstrated that HFNC can generate a flow-dependent positive airway pressure (P_aw_), which is proposed to be the main contributor to the improvement in oxygenation and lung volume using HFNC over conventional oxygen therapy^17–19^. However, limited data^20–22^ did not show clinically relevant changes in P_aw_ and end-expiratory lung volume (EELV) during high-flow tracheal (HFT) oxygen therapy via tracheostomy that might be caused by the different mechanisms of action during HFT compared with HFNC^10^. Additionally, controversial results were reported for the impact of HFT on oxygenation compared with T-piece^21,22^. These findings may be the major reasons for the limited use of HFT in tracheostomized patients.

In the present study, we modified the HFT system by adding a resistor with a physiological level of resistance to the expiratory port of the interface. HFT was delivered via tracheostomy in a bench model simulating spontaneous breathing, and thereafter, in pigs before and after the induction of mild lung injury. We primarily aimed to test whether the modified HFT could induce elevations of P_aw_ and EELV. As secondary study endpoints, we assessed the effects of modified HFT on inspiratory effort, work of breathing, lung stress, ventilation and gas exchange.

## Methods

### Modification of the HFT Interface

**Figure 1:**
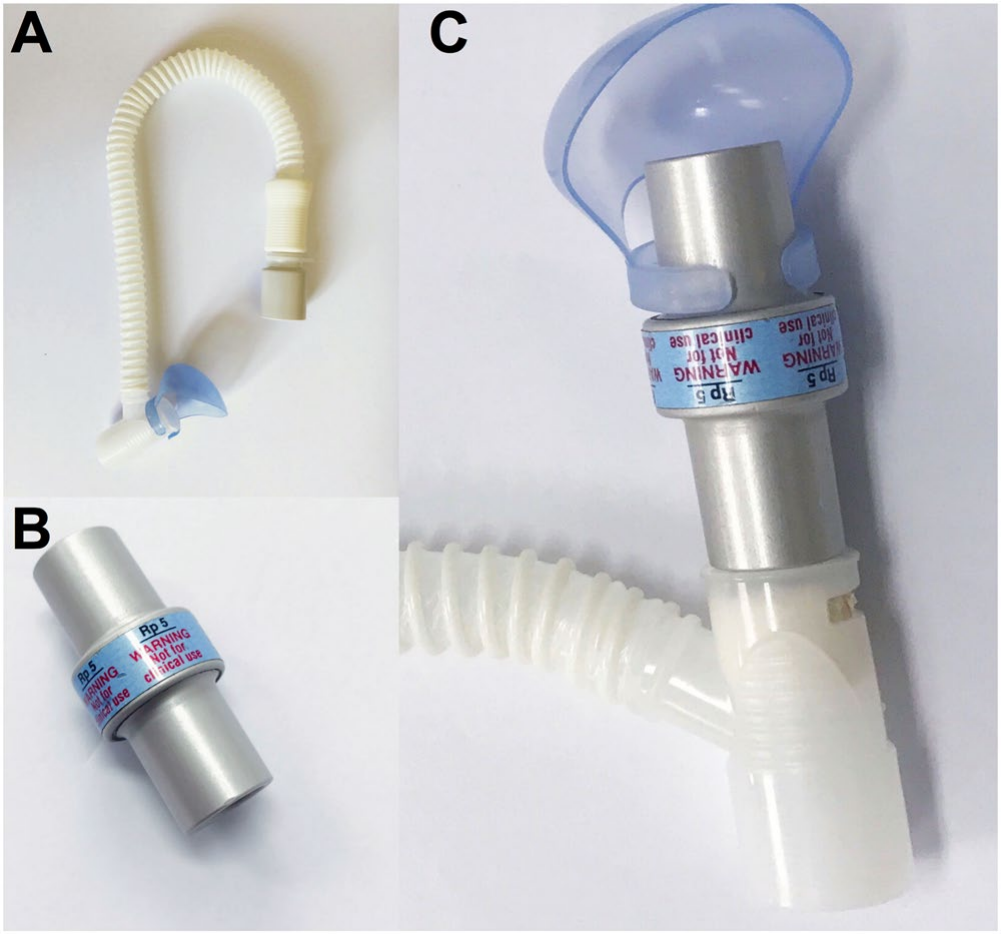
Standard and modified high-flow tracheal interface. (**A**) standard high-flow tracheal oxygen therapy interface; (**B**) test lung resistor (5 cm H_2_O/L/s); (**C**) modified interface by connecting the resistor to the expiratory port of the standard interface.

A 5-cm H_2_O/L/s resistor (Michigan Instruments, Grand Rapids, MI, USA) was connected to the expiratory port of an HFT interface (OPT870, Fisher & Paykel Healthcare, Auchland, New Zealand) (Fig. 1). During the study, HFT was delivered via the standard or modified interface using an AIRVO 2 device (Fisher & Paykel Healthcare, Auchland, New Zealand) and the manufacturer’s standard assembly composed of a heated breathing circuit and an auto-fill humidification chamber (900PT501, Fisher & Paykel Healthcare, Auchland, New Zealand).

### Bench experiment

Details of methods in the bench experiment are provided in Supplementary File.

A two-chamber Michigan test lung (Model 5600i, Michigan Instruments, MI, USA) was used to simulate spontaneous breathing, as previously described by Thille and coworkers^23^. Normal, strong and very strong inspiratory drives were simulated by setting the tidal volume (V_T_) at 300, 600 and 900 mL with peak inspiratory flows of 25, 50 and 75 L/min, respectively. The respiratory rate (RR) was set at 15 breaths/min to minimize the risk of air trapping, and no PEEP was used. Two levels of compliance were set to simulate a normal lung (60 mL/cm H_2_O) and a mild-injured lung (40 mL/cm H_2_O)^24^. Thus, six conditions were established with different inspiratory drives (normal, strong and very strong) and respiratory system compliances (normal and injured lung).

Under each condition, HFT was delivered via an 8.0 ID tracheostomy tube (Smiths Medical International Ltd, Kent, UK) using the standard and modified interface, and the flow rate was incrementally adjusted to 10, 20, 30, 40, 50 or 60 L/min with the HFT setting at inspired fraction of oxygen (F_IO2_) 0.21 and temperature of 37 °C.

The bench system was equilibrated for 10 min at each HFT flow level. A 6-French catheter (GE Healthcare, Helsinki, Finland) was inserted at 1 cm proximal to the end of the tracheostomy tube to measure the P_aw_. Pressure within the test lung (breathing chamber) was also measured by positioning the pressure transducer at the opening of test lung, defined as intrapulmonary pressure.

### Animal study

The animal study was approved by the Ethical Committee for Experimental Studies at Beijing Neurosurgical Institute, Beijing, China. All animal procedures were performed in accordance with the recommendations of the Guide for the Care and Use of Laboratory Animals of the National Institutes of Health. Detailed methods of the animal study are presented in Supplementary File.

Six healthy female pigs [Bama, weight: 38 to 45 kg (mean ± SD, 42 ± 3 kg), age: 11 to 13 months (mean ± SD, 12 ± 1 months)] were anesthetized via intramuscular ketamine (10 mg kg^−1^) and xylazine (1 mg kg^−1^). The animals were placed in the supine position on a thermo-controlled operation table to maintain rectal temperature at approximately 37 °C. A tracheostomy was performed, and an 8.0 ID tube (Smiths Medical International Ltd, Kent, UK) was placed. Mechanical ventilation was initiated in a pressure support (PS) mode with PS 10 cm H_2_O, PEEP 5 cm H_2_O and F_IO2_ 0.4. The pulse oxygen saturation and partial pressure of end-tidal carbon dioxide (P_ET_CO_2_) was monitored (BeneView T5, Mindray, Shenzhen, China). During the study, propofol (10 mg kg^−1^ h^−1^) and fentanyl (0.05 mg kg^−1^ h^−1^) were continuously infused to provide sedation and analgesia, minimizing suffering. P_aw_ was measured by inserting a 6-French catheter at 1 cm proximal to the end of the tracheostomy tube. An esophageal balloon catheter (Cooper: LOT 177405, Cooper Surgical, USA) was inserted for esophageal pressure (P_es_) measurement. The position of the balloon was confirmed by Baydur’s occlusion test^25^.

Electrical impedance tomography (EIT) monitoring (PulmoVista 500; Dräger Medical GmbH, Lübeck, Germany) was set up using a dedicated belt with 16 electrodes placed just below the axilla and one reference electrocardiogram electrode placed at the right lead leg. The images were continuously recorded at 40 Hz. Data were downloaded and analyzed off-line using a dedicated software (Dräger EIT Data Analysis Tool 6.3, Lübeck, Germany).

T-piece oxygen and HFT were delivered in the six animals before and after the induction of mild lung injury by surfactant depletion. Warmed normal saline (5 mL/kg at 37–39 °C) was instilled into the tracheostomy tube and then drained by gravity. Lavage was repeated until the partial pressure of oxygen in arterial blood (P_aO2_) to F_IO2_ (P_aO2_/F_IO2_) ratio was lower than 300 for 30 min^26^.

Before each investigation in the normal and injured lung model, the animal was mechanically ventilated in the PS mode. Propofol and fentanyl were titrated to maintain the absence of limb movement but adequate and stable spontaneous breathing for at least 30 min. Then, the animal was weaned from mechanical ventilation, and the following three tracheal oxygen treatments were performed in a random crossover fashion without washout period, lasting 20 min each:

1. Humidified T-piece oxygen
2. HFT via standard interface
3. HFT via modified interface

Humidified T-piece oxygen was delivered using an Oxyflo^TM^ system composed of an RT308 circuit and MR850 heated humidifier (Fisher & Paykel Healthcare, Auchland, New Zealand) at flow rate 10 L/min and temperature 37 °C. HFT was delivered using the same system mentioned in the bench experiment. HFT was set at flow rate 40 L/min, F_IO2_ 0.4 and temperature of 37 °C.

Propofol and fentanyl were not adjusted during each sequence of tests. At the end of study, the animals were sacrificed by intravenous infusion of 20 ml 10% potassium chloride under deep anesthesia.

### Data collection and measurements

Detailed methods of measurements are also provided in Supplementary File.

In the bench experiment and the animal study, pressures were measured by pressure transducers (KT 100D-2, Kleis TEK di CosimoMicelli, Italy, range:+/− 100 cmH_2_O) connected to an ICU-Lab Pressure Box (ICU Lab, KleisTEK Engineering, Bari, Italy) by 80 cm rigid tube lines. Flow tracings were continuously collected by a heated Fleisch pneumotachograph (Vitalograph Inc, Lenexa, KS, USA) placed between the high-flow tracheal (HFT) oxygen interface and the tracheostomy tube. Pressure and flow signals were displayed continuously and saved (ICU-Lab 2.5 Software Package, ICU Lab, KleisTEK Engineering, Bari, Italy) in a laptop for further analysis, at a sample rate of 200 Hz.

In the animal study, at the end of each tested phase (T-piece, HFT or modified HFT), hemodynamic data (HR and MAP), P_ET_CO_2_, P_aO2_ and partial pressure of carbon dioxide in arterial blood (P_aCO2_) were collected. The alveolar dead space fraction was calculated^27^.

Pressure and flow tracings in the last minute at each phase were analyzed, and the following parameters were collected:

1. The mean P_aw_ during either the inspiratory or expiratory phase^28^;
2. The peak inspiratory and expiratory flow rate (PIF and PEF);
3. The inspiratory V_T_ integrated by flow tracing, and RR and minute ventilation (MV);
4. The P_es_ swing during inspiration (∆P_es_)^29,30^;
5. The inspiratory and expiratory airway resistance estimated at flow rate of 200 mL/s using the method introduced by Mead *et al*.^31^ as follows:
  $${\rm{R}}=\frac{({{\rm{P}}}_{{\rm{0}}}-{\rm{P}})\frac{{\rm{V}}}{{\rm{C}}}}{{\rm{V}}^{\prime} }$$
  where R is the resistance, P_0_ is the P_es_ at the start of inspiratory and expiratory flow, V is the instantaneous volume integrated from flow, C is the dynamic compliance obtained for the same breath as the ratio of V_T_ to ∆P_es_, and V‘ is the instantaneous flow rate (=0.2 L/s);
6. The intrinsic PEEP that was equal to difference in P_es_ between onset of decrease of P_es_ and the start of inspiratory flow^29,30^;
7. The per-breath pressure time product (PTP) and the averaged PTP over a minute (PTP_min_) derived from P_es_ tracing^18^. The per-breath PTP was derived by integrating the area of the P_es_ waveform during inspiration of each breath in the last minute. PTP_min_ was calculated as the sum of per breath PTP in the last minute;
8. The dynamic end-inspiratory and end-expiratory transpulmonary pressure (P_L_) that were measured as the difference between P_aw_ and the absolute P_es_ measured at the end of inspiration and end of expiration (all at zero flow), And the driving transpulmonary pressure (∆P_L_) was calculated as the difference between end-inspiratory and end-expiratory P_L_^29,30^;
9. F_IO2_ estimated by the sum of fresh gas volume and room air entrainment as follows^32^:

F_IO2_ during T-piece oxygen was estimated as:

$${{\rm{F}}}_{{\rm{IO2}}}=\frac{({\rm{Ti}}\times 167\times 1.0)+({{\rm{V}}}_{{\rm{T}}}-{\rm{Ti}}\times {\rm{167}}\times 1.0)\times 0.21}{{{\rm{V}}}_{{\rm{T}}}}$$

where Ti is inspiratory time (s), 167 represents T-piece oxygen flow rate (10 L/min = 167 mL/s), and 0.21 represents oxygen concentration in air.

Actual F_IO2_ during HFT was estimated as:

$${{\rm{F}}}_{{\rm{IO2}}}=\frac{({\rm{Ti}}\times 667\times 0.40)+({{\rm{V}}}_{{\rm{T}}}-{\rm{Ti}}\times {\rm{667}}\times 0.40)\times 0.21}{{{\rm{V}}}_{{\rm{T}}}}$$

where 667 represents HFT flow rate used in the present study (40 L/min = 667 mL/s) and 0.40 represents set F_IO2_ during HFT.

And the P_aO2_/F_IO2_ ratio was also calculated.

In off-line EIT analysis, we defined the thoracic cross-section using a matrix of 32 × 32 pixels. The dorsal 8 × 32 pixels of this matrix were discarded because no lung was contained in this region of the pig’s anatomy^33,34^. The remaining 24 × 32 pixels were defined as the global region of interest (ROI), which were further evenly divided into the ventral ROI (non-dependent lung region), middle ROI and dorsal ROI (dependent lung region). EIT measurements were collected in the last minute of each phase, including the following:

1. Considering T-piece as the reference value, global and regional changes in EELV (∆EELV) during HFT via the two interfaces were evaluated as the respective change in end-expiratory impedance multiplied by the ratio between V_T_ measured by flow integration (in mL) and the global tidal impedance change (in absolute unit)^18,19^;
2. The regional distribution of tidal ventilation in the three ROIs was collected. The center of ventilation (COV) was calculated as the percentage of tidal ventilation distributed to the dorsal ROI in the global ROI^35^. The higher the COV, the more tidal ventilation is distributed to the dependent lung region.

### Statistical analysis

Normally distributed variables were presented as the means ± SD, and non-normally distributed variables were reported as the medians (25th to 75th percentile).

In the bench experiment, two-way analysis of variance (ANOVA) with repeated-measures was used to compare the P_aw_ and resistance across different HFT flow levels (10 to 60 L/min) as well as between the two HFT interfaces (standard and modified). A post hoc pairwise comparison was performed using the Bonferroni correction.

During modified HFT, the P_aw_ and flow rate were fitted using the following power equation:

$${{\rm{P}}}_{{\rm{aw}}}={\rm{a}}\times {{\rm{Flow}}}^{b}+c$$

The flow-P_aw_ curve was fitted using the Levenberg-Marquardt iterative algorithm, which was set to run until the change in the sum of squared residuals was lower than 10^−8^. The coefficient of determination (*R*^2^) was calculated.

For the modified interface, a multiple stepwise linear regression was performed to find the potential determinants of the mean expiratory P_aw_. The covariates that were entered into the model included the quadratic element of flow rate (flow^2^), set compliance of the breathing chamber and expiratory resistance.

In the animal study, differences in variables across different tracheal oxygen treatments (T-piece, HFT and modified HFT) were compared by one-way ANOVA with repeated-measures or by Friedman test, as appropriate. Post hoc pairwise comparisons were performed using the Bonferroni correction. The correlations were analyzed using the Pearson coefficient (*R*).

Analyses were conducted using SPSS 20.0 (SPSS, Chicago, Illinois, USA). A *p* < 0.05 was considered statistically significant.

## Results

### Bench experiment

The bench experiment results are detailed in Supplementary File.

Compared with the HFT, the modified HFT generated significantly higher mean expiratory P_aw_ at each flow rate level, and significantly higher mean inspiratory P_aw_ at flow rates from 30 to 60 L/min (*p* < 0.05, Table 1). For the modified HFT, either inspiratory or expiratory P_aw_ increased as a power function of flow rate (see the Supplementary Fig. S1).

**Table 1:** Airway pressure and resistance during high-flow tracheal oxygen in the bench experiment.

	HFT flow rates (L/min)						*p* among flow rates
	10	20	30	40	50	60	
**Mean inspiratory P**_**aw**_ **(cmH**_**2**_ **O)**							
Standard interface	−0.9 ± 0.6	−1.1 ± 0.8	−0.8 ± 0.7	−0.7 ± 0.7	−0.6 ± 0.8	−0.4 ± 0.8	0.621
Modified interface	−1.1 ± 0.8	−0.8 ± 0.8	−0.3 ± 0.9	0.6 ± 1.1	2.1 ± 1.3	4.0 ± 1.4	<0.001
*p* between interfaces	0.130	0.153	0.003	<0.001	<0.001	<0.001	
**Mean expiratory P**_**aw**_ **(cmH**_**2**_ **O)**							
Standard interface	0.5 ± 0.3	0.6 ± 0.3	0.8 ± 0.3	1.0 ± 0.3	1.2 ± 0.3	1.5 ± 0.3	<0.001
Modified interface	0.9 ± 0.5	1.6 ± 0.6	2.6 ± 0.6	3.8 ± 0.6	5.3 ± 0.6	7.0 ± 0.7	<0.001
*p* between interfaces	0.004	<0.001	<0.001	<0.001	<0.001	<0.001	
**Inspiratory resistance (cmH**_**2**_ **O/L/s)**							
Standard interface	3.8 ± 0.4	3.8 ± 0.6	3.9 ± 0.7	4.1 ± 0.6	4.1 ± 0.5	4.4 ± 0.6	0.389
Modified interface	4.5 ± 0.3	4.6 ± 0.6	5.5 ± 0.5	6.3 ± 0.2	6.2 ± 0.9	5.5 ± 1.7	0.002
*p* between interfaces	0.014	0.001	0.002	< 0.001	< 0.001	0.166	
**Expiratory resistance (cmH**_**2**_ **O/L/s)**							
Standard interface	6.2 ± 1.0	6.2 ± 0.9	6.4 ± 0.7	6.5 ± 0.9	6.6 ± 0.9	6.6 ± 0.8	0.905
Modified interface	7.6 ± 1.5	7.6 ± 1.1	8.7 ± 1.1	9.6 ± 1.1	10.5 ± 1.1	11.9 ± 1.3	<0.001
*p* between interfaces	0.009	0.002	<0.001	<0.001	<0.001	<0.001	

HFT: high-flow tracheal oxygen; *P*_*aw*_ airway pressure.

Data are shown as mean ± standard deviation.

Compared with the HFT, the modified HFT significantly increased expiratory resistance at each flow rate level (*p* < 0.05) with the maximal change from 6.6 ± 0.9 cm H_2_O/L/s to 11.9 ± 1.3 cm H_2_O/L/s at flow rate of 60 L/min. Although there was also a statistical significance in inspiratory resistance during modified HFT, the magnitude was relatively minor. (Table 1).

For the modified HFT, the covariates that determined the mean expiratory P_aw_ included the flow^2^ and expiratory resistance (*R*^2^ = 0.963, see the Supplementary Table S1).

### Animal study

After a normal saline lavage of the lungs, the P_aO2_/F_IO2_ ratio decreased from 352 ± 77 to 228 ± 45 (see the Supplementary Table S2). All the animals tolerated tracheal oxygen treatments during the study.

### Effects of Modified HFT on P_aw_ and Resistance

**Figure 2:**
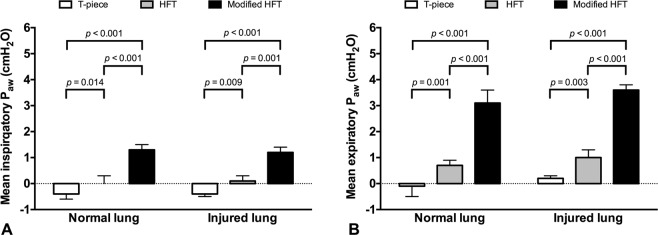
Airway pressure (P_aw_) during T-piece, high-flow tracheal (HFT) oxygen and modified HFT. In both normal and injured lung models, modified HFT induced significantly higher inspiratory (**A**) and expiratory (**B**) P_aw_ compared with either T-piece or HFT. Data are presented as means and standard deviations, and *p* values in pairwise comparisons are also shown.

**Figure 3:**
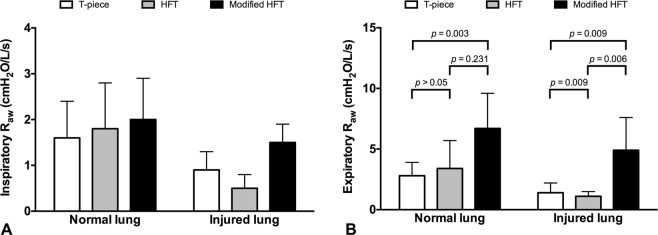
Airway resistance during T-piece, high-flow tracheal (HFT) oxygen and modified HFT. There was not a significant difference in inspiratory resistance (**A**) among the three tracheal oxygen treatments (*p* = 0.484 in normal lung group and *p* = 0.056 in injured lung group). Expiratory resistance (**B**) significantly increased during modified HFT compared with T-piece. Data are presented as means and standard deviations, and *P* values in pairwise comparisons are also shown.

In both normal and injured lung models, modified HFT induced significantly higher inspiratory and expiratory P_aw_ compared with either T-piece or HFT (*p* < 0.05, Fig. 2). Although there was an increasing tendency in inspiratory resistance via modified HFT, no significant difference was found among the three treatment groups in both lung conditions (Fig. 3A). Expiratory resistance significantly increased during modified HFT (*p* < 0.05, Fig. 3B) to a mean value of 6.7 ± 2.9 (range: 4.1–11.6) and 4.9 ± 2.7 (1.9–9.2) cm H_2_O/L/s in the normal and injured lung model, respectively. There was a significant correlation between expiratory P_aw_ and resistance (*R* = 0.577, *p* < 0.001).

During modified HFT, a significant decrease was found in PEF in both models and in PIF in the injured lung model (*p* < 0.05). There was a decreasing tendency in PIF in the normal lung model, but it was not statistically significant (Table 2).

**Table 2:** Effects of modified high-flow tracheal oxygen on ventilation, inspiratory efforts, transpulmonary pressure and work of breathing

	Normal				Injured			
	T-piece	HFT	Modified HFT	*p*	T-piece	HFT	Modified HFT	*p*
RR (breaths/min)	28 ± 13	30 ± 13	26 ± 14	0.149	48 ± 4	49 ± 4	44 ± 5^a^	0.011
Ti (s)	0.8 ± 0.3	0.7 ± 0.2	0.8 ± 0.2	0.116	0.5 ± 0.1	0.5 ± 0.1	0.6 ± 0.1	0.694
Te(s)	1.8 ± 0.9	1.6 ± 0.9	1.6 ± 1.0	0.109	0.8 ± 0.2	0.7 ± 0.1	0.7 ± 0.1	0.694
V_T_ (mL)	228 ± 36	216 ± 44	231 ± 40	0.475	195 ± 27	180 ± 24	190 ± 23	0.159
MV (L/min)	6.2 ± 3.0	6.2 ± 1.9	5.5 ± 2.3	0.203	9.2 ± 1.2	8.7 ± 1.0	8.2 ± 1.2	0.194
PIF (mL/s)	504 ± 146	487 ± 153	432 ± 81	0.209	632 ± 127	572 ± 128^a^	501 ± 130^a,b^	0.005
PEF (mL/s)	593 ± 104	536 ± 114^a^	462 ± 52^a,b^	0.047	526 ± 31	480 ± 33^a^	445 ± 52^a,b^	0.008
Intrinsic PEEP (cmH_2_O)	0.2 ± 0.2	0.1 ± 0.2	0.2 ± 0.2	0.086	0.0 ± 0.0	0.0 ± 0.0	0.0 ± 0.1	0.605
∆P_es_ (cmH_2_O)	5.7 ± 2.3	5.0 ± 2.1	5.9 ± 2.1	0.131	6.2 ± 1.1	6.4 ± 1.8	6.8 ± 2.2	0.372
∆P_L_ (cmH_2_O)	5.3 ± 2.2	4.7 ± 2.0	5.7 ± 1.5	0.146	6.0 ± 1.8	6.4 ± 1.9	7.1 ± 2.3	0.078
PTP (cmH_2_O × s)	3.9 ± 2.1	3.1 ± 1.3	3.6 ± 1.3	0.238	2.8 ± 0.8	2.4 ± 0.6	2.9 ± 0.9	0.091
PTP_min_ (cmH_2_O × s/min)	93.4 ± 38.4	83.0 ± 30.6	82.4 ± 36.6	0.101	133.6 ± 42.5	117.4 ± 33.7	126.5 ± 39.9	0.176

HFT: high-flow tracheal oxygen; MV: minute ventilation; PEEP: positive end-expiratory pressure; PEF: peak expiratory flow; PIF: peak inspiratory flow; PTP: per-breath pressure time product; PTP_min_: averaged pressure time product over a minute; RR: respiratory rate; Ti: inspiratory time; Te: expiratory time; V_T_: tidal volume; ∆P_es_: esophageal pressure swing during inspiration; ∆P_L_: driving transpulmonary pressure.

Data are shown as mean ± standard deviation.

^a^Significantly different compared with T-piece.

^b^Significantly different compared with HFT.

No obvious intrinsic PEEP was identified during each tracheal oxygen treatment (Table 2).

### Effects of Modified HFT on Lung Volume, Ventilation and Distribution

**Figure 4:**
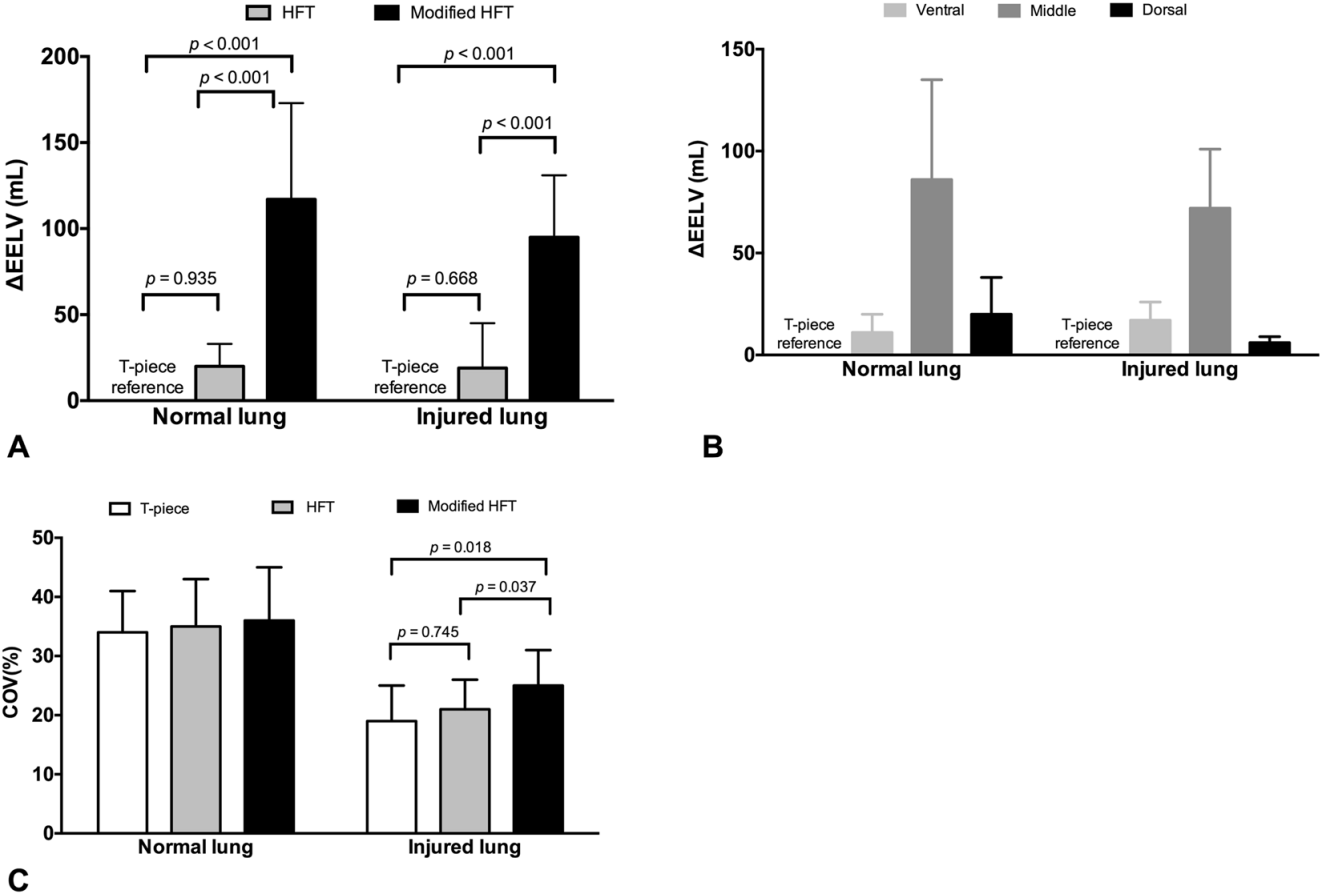
Changes in end-expiratory lung volume (∆EELV), distribution of ∆EELV and center of ventilation (COV) during high-flow tracheal (HFT) oxygen and modified HFT. Compared with the HFT and T-piece, the modified HFT induced significant global ∆EELV (**A**). ∆EELV was mainly distributed to the middle region of interest (**B**). In the normal lung model, there was no significant difference in COV among the three treatments (*p* = 0.357), but in the injured lung model, a significantly higher COV was found via the modified HFT compared with T-piece (*p* = 0.018) (**C**). Data are presented as means and standard deviations, and *p* values in pairwise comparisons are shown.

Global ∆EELV increased significantly with the modified HFT when compared to HFT in both lung conditions, normal and injured, respectively (*p* < 0.05, Fig. 4A). ∆EELV was mainly distributed to the middle ROI (Fig. 4B). Furthermore, ∆EELV positively correlated with expiratory P_aw_ (*R* = 0.766, *p* < 0.001).

No significant differences were found in V_T_, Ti and expiratory time (Te) among the three treatments, whereas RR decreased only, but significantly in the modified HFT group for the injured lung model (*p* = 0.011, Table 2). However, there was no significant change in MV observed among any treatment groups and/or between the two lung conditions.

No significant difference in COV was observed among the three treatment groups with the only exception of a higher COV for the modified HFT group compared to the HFT group (*p* = 0.037) and the T-piece group (*p* = 0.018) in the injured lung condition (Fig. 4C).

### Effects of Modified HFT on Inspiratory Effort, ∆P_L_ and Work of Breathing

There was no significant difference in either ∆P_es_, ∆P_L_, per-breath PTP or PTP_min_ among the three treatments (Table 2).

### Effects of Modified HFT on Gas Exchange and Hemodynamics

F_IO2_ and P_aO2_ during T-piece were significantly higher than those during HFT and modified HFT (Table 3). P_aO2_/F_IO2_ ratios were unchanged among the three treatment groups in the normal lung model but increased significantly during modified HFT compared to those during HFT and T-piece in the injured lung model (*p* = 0.038, Table 3).

**Table 3:** Effects of modified high-flow tracheal oxygen on gas exchange and hemodynamics.

	Normal			*p*	Injured			*p*
	T-piece	HFT	Modified HFT		T-piece	HFT	Modified HFT	
FiO_2_	0.77 ± 0.15	0.40 ± 0.02^a^	0.41 ± 0.01^a^	0.002	0.79 ± 0.13	0.41 ± 0.02^a^	0.40 ± 0.02^a^	0.001
PaO_2_ (mmHg)	310 ± 120	173 ± 47^a^	185 ± 37^a^	0.019	214 ± 89	113 ± 32^a^	125 ± 36^a^	0.010
PaO_2_/FiO_2_ ratio	412 ± 164	432 ± 119	457 ± 98	0.228	279 ± 115	276 ± 79	308 ± 77^a,b^	0.038
P_ET_CO_2_ (mmHg)	39 ± 4	39 ± 4	40 ± 5	0.350	38 ± 5	40 ± 5	38 ± 3	0.307
PaCO_2_ (mmHg)	50 ± 5	50 ± 2	50 ± 2	0.968	57 ± 3	53 ± 5	53 ± 2	0.316
Alveolar dead space fraction	0.22 ± 0.10	0.21 ± 0.10	0.19 ± 0.07	0.701	0.33 ± 0.05	0.24 ± 0.07	0.28 ± 0.07	0.075
Mean arterial pressure (mmHg)	119 ± 14	114 ± 13	116 ± 11	0.238	111 ± 25	112 ± 13	116 ± 16	0.737
Heart rate	77 ± 21	68 ± 11	63 ± 8	0.371	73 ± 7	72 ± 7	66 ± 9^a^	0.041

FiO_2_: fraction of inspired oxygen; PaO_2_: partial pressure of oxygen in arterial blood; PaCO_2_: partial pressure of carbon dioxide in arterial blood,; P_ET_CO_2_: partial pressure of end-tidal carbon dioxide.

Data are shown as mean ± standard deviation.

^a^Significantly different compared with T-piece.

^b^Significantly different compared with HFT.

There was no significant difference in MAP and HR among the three treatments, with the exception of a significantly decreased HR in the modified HFT group compared to the T-piece group in the injured lung model (*p* = 0.041, Table 3).

## Discussion

In the present study, we modified the HFT system by adding a 5 cm H_2_O/L/S resistor to the expiratory port of the standard interface. The effect of modified HFT on P_aw_ was first reported in a bench model. Then, the performance of modified HFT via tracheostomy was evaluated in pigs with normal and mildly injured lungs. Our results demonstrated that the modified HFT generated flow-dependent positive P_aw_ and, consequently, an increase in EELV, which might be the main reasons for the improvement in ventilation homogeneity and oxygenation. Meanwhile, the increase in expiratory resistance was within an acceptable range and did not significantly affect the inspiratory efforts, lung stress and work of breathing.

Supplemental oxygen therapy is one of the most commonly used treatment modalities in critically ill patients. Recent evidence suggested that when compared with standard oxygen therapy, HFNC improved oxygenation and respiratory mechanics^17–19^. These improvements were proposed to be mainly due to the elevations in P_aw_ and EELV, which might have resulted from increased expiratory resistance as the high inward flow encounters the nasal airway^36,37^. However, this encountered resistance is diminished after tracheostomy because the larynx and upper airway are bypassed^7,8^. Accordingly, limited investigations revealed that no clinically significant positive P_aw_ and EELV effects were found during HFT via tracheostomy when compared with T-piece oxygen, although oxygenation was improved^20–22^. These findings somewhat resembled the delivery of HFNC with opened mouth, during which the extra expiratory resistance vanished, and the P_aw_ effect disappeared^12,15,16^. Therefore, we speculated that adding a resistor to the expiratory port of the HFT interface might mimic the nasal resistance during expiration, thus inducing a positive P_aw_ effect and consequently elevating EELV. Our results confirmed this hypothesis. During modified HFT via tracheostomy, a marked P_aw_ effect was found in the bench experiment and animal study, and ∆EELV significantly increased in the animal study, with resistance remaining within an acceptable range. These results indicated the efficacy of the modification.

Several safety considerations must be assessed when using the modified HFT. The first concern is the extent of resistance induced by the modification. We added a physiological level resistor, i.e., 5 cm H_2_O/L/s^38^, to the expiratory port of the interface (Fig. 1). Compared with the HFT, although expiratory resistance increased significantly via the modified interface in both bench and animal tests, it remained within an acceptable range, with mean values at different flow rates ranging from 7.6 ± 1.5 to 11.9 ± 1.3 cm H_2_O/L/s in the bench experiment (Table 1), and 6.7 ± 2.9 and 4.9 ± 2.7 cm H_2_O/L/s in the animal model with normal and injured lungs, respectively (Fig. 3B). These resistance levels were also comparable to those obtained during HFNC in the bench study (10.15 ± 1.37 cm H_2_O/L/s)^39^ and in patients recovering from acute respiratory failure (median [25th to 75th percentile] of 6.7 [5.6–8.8] cm H_2_O/L/s at 40 L/min flow rate)^40^. Meanwhile, the inspiratory resistance also slightly increased in the modified HFT. But the increment is within physiological range. The reason for the elevation of inspiratory resistance might be due to the increase of end-expiratory P_aw_ during the modified HFT, which is the component in the calculation of resistance introduced by Mead, *et al*.^31^. The second safety concern is whether the elevated resistance affects inspiratory efforts and lung stress. Strong inspiratory efforts with collateral elevation of resistance could have resulted in high P_L_, i.e., high lung stress, which could aggravate lung injury. ∆P_es_ is a validated measurement of inspiratory effort^29,30^. We used the ∆P_L_ to avoid the influence of absolute P_es_ on the measurements of inspiratory and expiratory P_L_. No significant differences in ∆P_es_ and ∆P_L_ were found among the three oxygen therapy modalities in our animal study. Moreover, ∆P_es_ and ∆P_L_ via the modified HFT in our pig model with lung injury (6.8 ± 2.2 and 7.1 ± 2.3 cm H_2_O, respectively) were comparable to those in acute lung injured patients receiving HFNC at the same flow rate reported by Mauri *et al*. (8.0 and 4.3 cm H_2_O)^18^ and Delorme *et al*. (7.6 cm H_2_O)^40^. These results suggested that the elevation in resistance induced by the modification was less likely to increase the inspiratory efforts and lung stress above an injurious level. The third concern is the potential effect of increased resistance to the work of breathing. No significant elevation was found in either per-breath PTP or PTP_min_ during modified HFT. Our results for tracheal oxygen via tracheostomy (2.4 ± 0.6 to 3.9 ± 2.1 cm H_2_O × s for per-breath PTP and 82.4 ± 36.6 to 133.6 ± 42.5 cm H_2_O × s/min for PTP_min_, Table 2) were lower than those reported in patients during oxygen therapy via facial mask and HFNC (7.4 to 9.5 cm H_2_O × s for per-breath PTP and 154.8 to 216.3 cm H_2_O × s/min for PTP_min_)^18^, which suggests a decline in the work of breathing via tracheostomy. Meanwhile, modified HFT did not result in intrinsic PEEP in all conditions. In summary, for safety considerations, modified HFT increased expiratory resistance to an acceptable level and did not significantly influence the inspiratory efforts, lung stress and work of breathing.

In the bench and animal studies, we reported a P_aw_ effect via modified HFT, approximately 4 cm H_2_O at the flow of 40 L/min, which was comparable to that reported in bench experiments^12^ and adult patients^13–17^ with HFNC. Previous HFNC studies showed that the P_aw_ effect was determined by flow, with a linear^13,15,16^ or quadratic^12,36^ relationship between the P_aw_ and HFNC flow rate. In the bench experiment with modified HFT, we found that the P_aw_ and flow rate fitted well with a power function curve (see the Supplementary Fig. E1), and a multiple linear regression analysis identified the expiratory resistance as another determinant of the P_aw_ effect (see Supplementary File Table S1). Moreover, as the flow rate increased, an elevation in resistance was induced only via modified HFT, but not HFT. In the animal study, a direct correlation was found between the expiratory P_aw_ and resistance. These results were in accordance with our hypothesis. It can be proposed that by adding a physiological level resistor to the expiratory port of the HFT interface, the high flow of air that was encountered increased resistance during expiration, which markedly induced the elevation of P_aw_.

As far as we know, due to the influence of V_T_, RR and inspiratory time, actual F_IO2_ is not stable during low-flow oxygen system, such as T-piece^10,32^. The HFT had an advantage of providing an accurate setting of F_IO2_^10^. In the studies comparing oxygenation during HFT and T-piece, F_IO2_ delivered by T-piece was usually estimated by the approximation of oxygen flow rate and physiological dead space^21,22^. In the present study, we used a pneumotachograph to measure the inspiratory flow rate, and actual F_IO2_ was calculated by the sum of fresh gas volume and room air entrainment as previously described^32^. This method could provide relatively accurate F_IO2_ measurement. A significantly higher F_IO2_ was found during T-piece oxygen than HFT, which was in accordance with the results presented by Corley *et al*.^21^. Our data suggested that oxygenation during T-piece should be interpreted with caution because of the unstable F_IO2_ delivery during low-flow oxygen system.

Our animal results preliminarily demonstrated some potential clinical benefits of the P_aw_ effect produced by modified HFT. ∆EELV, indicating an improvement in lung volume and a reduction of alveolar collapse, correlated directly to the mean expiratory P_aw_. The increase in P_aO2_/F_IO2_ ratio in the injured lung model might have largely resulted from the elevation in EELV. Additionally, the increase in ∆EELV in combination with unchanged V_T_ suggested a reduction in lung strain, indicating that there was a low risk in causing lung injury, e.g. hyperinflation. These findings are comparable to those reported in lung-injured patients receiving HFNC^18,19^. Finally, a slight but significant increase in COV in the injured lung model suggested a potential reduction in the stress generated by inhomogeneity between the dependent and non-dependent lung regions^18^.

There are limitations in the present study. First, in the animal study, we only tested a single HFT flow rate (40 L/min) and investigated the acute physiological responses (within 20 min of treatment) to the tracheal oxygen treatments without washout period; it was relatively difficult to maintain an optimal sedation level with stable spontaneous breathing and no agitation in the tested animal for an extended period of time. However, the flow rate chosen in the present study represented the low flow level used in the clinical studies of HFNC and HFT^15–18,20^, making it convenient to compare our results with previous reports. Additionally, our equilibrating time was likely enough for the main endpoints of P_aw_ and lung volume effects^21^. Second, although the measurement of resistance introduced by Mead *et al*. has been employed in clinical studies^32,41^, the use of dynamic compliance in the equation might overestimate the airway resistance, even though our measured expiratory resistance remained within an acceptable range. Third, we only calculated F_IO2_ using an equation based on the proportion of delivered fresh gas volume and entrainment volume room air, rather than direct measurement. This might have influenced the P_aO2_/F_IO2_ ratio results, especially during T-piece oxygen. Forth, in bench and animal experiment, we did not observe the effects of modified HFT on conditions with obstructive diseases. Although the rationale and physiology were recently discussed for the use of HFNC in stable chronic obstructive pulmonary disease^42^, high-flow oxygen therapy in a severe airway obstructive condition still remains to be clarified. Fifth, although portable continuous positive airway pressure (CPAP) devices are available (such as Boussignac^TM^ oxygen therapy device), they are not widely used^43^. Thus, we didn’t compare HFT with CPAP devices in our study.

## Conclusions

Our modified HFT with additional expiratory resistance generated clinically relevant flow-dependent P_aw_ and lung volume effects, which might be the main reasons for improvements in oxygenation and ventilation homogeneity. Meanwhile, inspiratory effort, lung stress and work of breathing remained within normal ranges. Our introduced modification provides an opportunity for potential improvements in the HFT instrument, which may be beneficial for oxygen therapy in tracheostomized patients. Clinical feasibility and safety require further investigation.

## Supplementary information

Supplementary information

## Data Availability

The datasets generated during and/or analyzed during the current study are available from the corresponding author on reasonable request.
