# Supplementary material for: Additional Expiratory Resistance Elevates Airway Pressure and Lung Volume during High-Flow Tracheal Oxygen via Tracheostomy
Source: Sci Rep. 2019 Oct 10;9:14542. doi: 10.1038/s41598-019-51158-0 (PMC6787229; doi:10.1038/s41598-019-51158-0)
Supplement: Supplementary file 1 — Supplementary information [file 41598_2019_51158_MOESM1_ESM.pdf]

Additional Expiratory Resistance Elevates Airway Pressure and Lung Volume during High-Flow Tracheal Oxygen via Tracheostomy

Guang-Qiang Chen, Xiu-Mei Sun, Yu-Mei Wang, Yi-Min Zhou, Jing-Ran Chen, Kun-Ming Cheng, Yan-Lin Yang & Jian-Xin Zhou*

All authors are affiliated with the Department of Critical Care Medicine, Beijing Tiantan Hospital, Capital Medical University, Beijing, China.

*corresponding. Jian-Xin Zhou MD, Department of Critical Care Medicine, Beijing Tiantan Hospital, Capital Medical University, No.119 South 4th Ring West Road, Fengtai District, 100070, Beijing, China. E-mail: zhoujx.cn@icloud.com.

Authors' contribution statements

Study concept and design: G.Q.C., X.M.S. and J.X.Z. Data acquisition: G.Q.C., X.M.S., Y.M.W., Y.M.Z., J.R.C. and J.X.Z. Analysis of data: G.Q.C., X.M.S., Y.M.Z., J.R.C. K.M.C., Y.L.Y. and J.X.Z. Manuscript preparation: G.Q.C., and J.X.Z. All authors reviewed the manuscript.

Acknowledgements

This study was supported by grants from the Beijing Municipal Administration of Hospital (ZYLX201502). The sponsor had no role in the study design, data collection, data analysis, data interpretation, or writing of the report.

Competing interests statement: G.Q.C., X.M.S. and J.X.Z. have applied for a patent on the modified interface of high-flow tracheal oxygen via tracheostomy interface. Other authors declare that they have no competing interest.

Contents

Detailed methods in the bench experiment

Detailed methods of the animal study

Detailed methods of measurements

Detailed bench experiment results

Figure S1. Relationship between airway pressure and flow rate with modified high-flow tracheal oxygen

Table S1. Multiple linear regression for determinants of mean expiratory airway pressure during modified high-flow tracheal oxygen

Table S2. Characteristics before and after the induction of lung injury

Detailed methods in the bench experiment

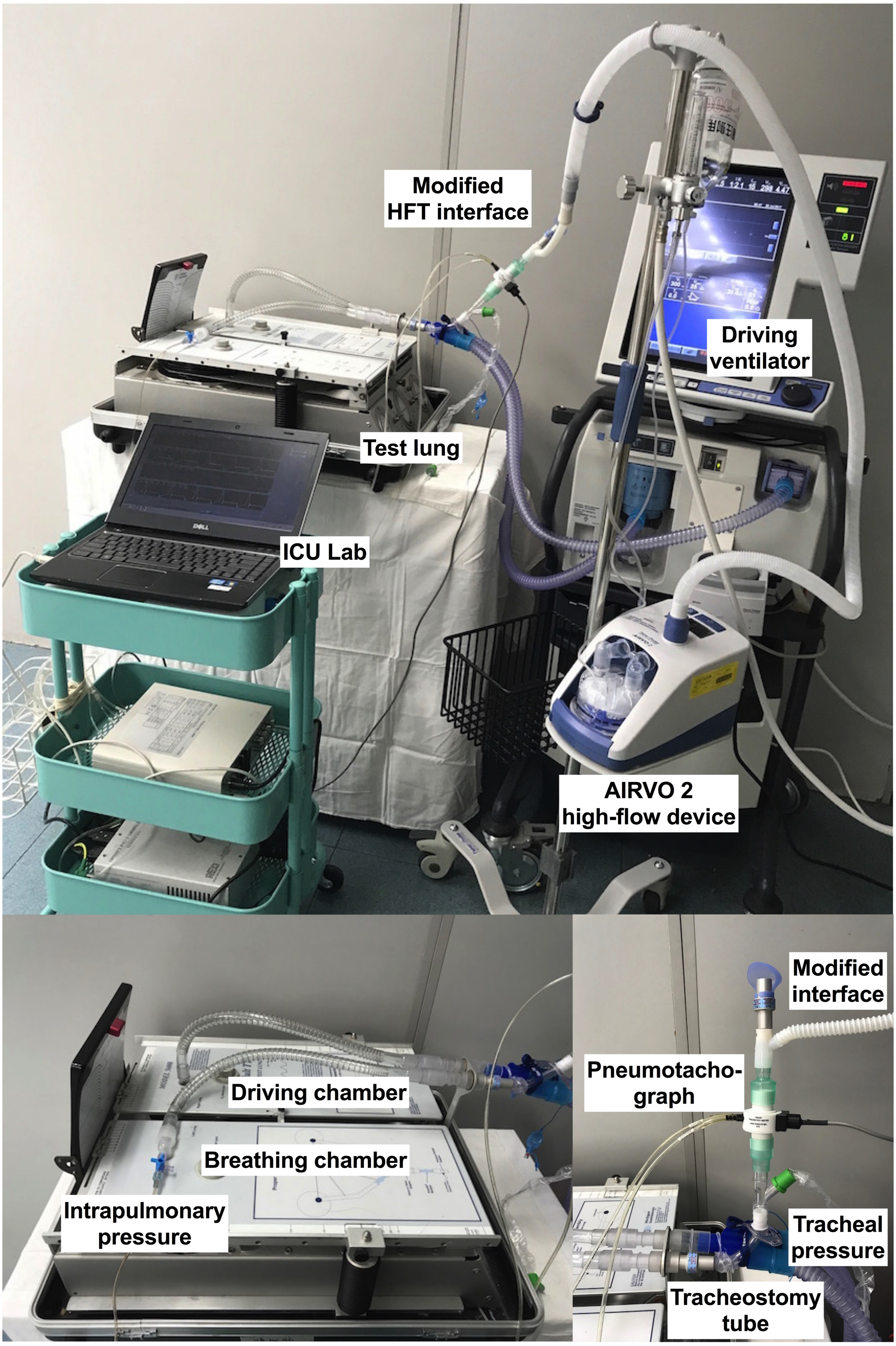

A two-chamber Michigan test lung (Dual Adult Training Test Lung, Model 5600i, Michigan Instruments, Grand Rapids, MI, USA) was used to simulate spontaneous breathing [S1]. The two chambers were linked by a rigid metal bridge. One chamber was connected to a driving ventilator

(Puritan Bennett™ 840, Covidien Co., Ltd., Tyco Healthcare International Trading, Shanghai, China) and the other chamber to the high-flow tracheal (HFT) interface via an 8.0 ID tracheostomy tube (Smiths Medical International Ltd, Kent, UK). The inflation of the driving chamber by the driving ventilator produced a negative pressure in the breathing chamber, which simulated inspiratory efforts. The driving ventilator was set as volume-controlled ventilation with decelerating flow. The tidal volume was set at 300, 600 and 900 mL with respective peak inspiratory flow of 25, 50 and 75 L/min to simulate normal, strong and very strong inspiratory drives [S1]. Respiratory rate was set as 15 breaths/min to minimize the risk of air trapping and no positive end-expiratory pressure (PEEP) was used. The breathing chamber was set to simulate a normal lung condition with a compliance of 60 mL/cmH2O and a mild lung injury condition with a compliance of 40 mL/cmH2O, all with resistance of 5 cmH2O/L/s [S2]. Therefore, the six conditions were simulated with different inspiratory drives (normal, strong and very strong) and respiratory system conditions (normal and injured lung). HFT was delivered via standard and modified interface by an AIRVO 2 device (Fisher & Paykel Healthcare, Auchland, New Zealand) and a manufacturer's standard assembly equipped with a heated breathing circuit and an auto-fill humidification chamber (900PT501, Fisher & Paykel Healthcare, Auchland, New Zealand). Under each condition, the HFT flow rate was incrementally adjusted to 10, 20, 30, 40, 50 or 60 L/min with the HFT setting as inspired fraction of oxygen (FiO_2) 0.21 and temperature 37 °C. The bench system was equilibrated for 10 min at each flow level. A 6-French catheter (GE Healthcare, Helsinki, Finland) was inserted at 1 cm proximal to the end of the tracheostomy tube to measure the airway pressure (P_{aw}). Pressure within the breathing chamber was also measured. All pressure measurements were performed by pressure transducers (KT 100D-2, Kleis TEK di CosimoMicelli, Italy, range: +/- 100 cmH_2O) connected to an ICU-Lab Pressure Box

(ICU Lab, KleisTEK Engineering, Bari, Italy) by 80 cm rigid tube lines. Flow was measured by a heated Fleisch pneumotachograph (Vitalograph Inc, Lenexa, KS, USA) placed between the HFT interface and the tracheostomy tube. Pressure and flow signals were displayed continuously and saved (ICU-Lab 2.5 Software Package, ICU Lab, KleisTEK Engineering, Bari, Italy) in a laptop for further analysis, at a sample rate of 200 Hz. The airway resistance was estimated using the method introduced by Mead et al [S3]. Inspiratory and expiratory resistance was measured separately at flow rate of 200 mL/s. Change in pressure within the breathing chamber was used to calculate the resistance:

$$R = [(P_0\text{-}P)\text{-}(\frac{V}{C})]/\dot{V}$$

where R is the resistance, P_0 is the pressure at the start of inspiratory or expiratory flow, V is the instantaneous volume integrated from flow, C is the compliance of the breathing chamber, and $\dot{V}$ is the instantaneous flow rate (= 0.2 L/s).

References

S1. Thille AW, Lyazidi A, Richard JC, Galia F, Brochard L. A bench study of intensive-care-unit ventilators: new versus old and turbine-based versus compressed gas-based ventilators. Intensive Care Med. 2009; 35: 1368-76.

S2. Lyazidi A, Thille AW, Carteaux G, Galia F, Brochard L, Richard JC. Bench test evaluation of volume delivered by modern ICU ventilators during volume-controlled ventilation. Intensive Care Med. 2010; 36: 2074-80.

S3. Mead J, Whittenberger JL. Physical properties of human lungs measured during spontaneous respiration. J Appl Physiol. 1953; 5: 779–96.

Detailed methods of the animal study

The animal study was approved by the Ethical Committee for Experimental Studies at Beijing Neurosurgical Institute, Beijing, China.

Preparation and instrumentation

Six healthy female pigs (Bama, weight: 38 to 45 kg) were anesthetized with intramuscular ketamine (0.3 mg/kg) and xylazine (0.3 mg/kg). The animals were placed in the supine position on a thermo-controlled operation table to maintain rectal temperature at approximately 37 °C. Femoral venous catheterization was performed for fluid and drug administration, and femoral arterial catheterization for invasive blood pressure monitoring and blood gas analysis sampling. An 8.0 ID tracheostomy tube (Smiths Medical International Ltd, Kent, UK) was placed and mechanical ventilation was initiated in a pressure support (PS) mode with PS 10 cmH_2O, positive end-expiratory pressure (PEEP) 5 cm H_2O and inspired oxygen fraction (FiO_2) 0.4. Pulse oxygen saturation and partial pressure of end-tidal carbon dioxide ($P_{ET}CO_2$) was monitored (BeneView T5, Mindray, Shenzhen, China). During the study, normal saline was intravenously infused at a rate of 5 mL/kg/h. The mean arterial pressure (MAP) was maintained above 65 mmHg. During the preparation and instrumentation, propofol and fentanyl were continuously infused to provide sedation and analgesia.

Airway pressure (P_{aw}) was measured by inserting a 6-French catheter at 1 cm proximal to the end of the tracheostomy tube. An esophageal balloon catheter (Cooper: LOT 177405, Cooper Surgical, USA) was inserted for esophageal pressure (P_{es}) monitoring. The position of the balloon was confirmed by standard occlusion test [S1].

Electrical impedance tomography (EIT) monitoring (PulmoVista 500; Dräger Medical GmbH, Lübeck, Germany) was setup using a dedicated belt with 16 electrodes placed at just below the

axilla and the one reference electrocardiogram electrode placed at the right lead leg. The images were continuously recorded at 40 Hz. Data were downloaded and off-line analyzed using a dedicated software (Dräger EIT Data Analysis Tool 6.3, Lübeck, Germany).

Lung injury model

The six animals were investigated before and after the establishment of a mild lung injury model, which was induced by surfactant depletion. Dosage of fentanyl was increased to diminish spontaneous breathings. Mechanical ventilation was switched to volume-controlled mode with constant flow, tidal volume (V_T) 8 mL/kg, respiratory rate (RR) 20 breaths/min, PEEP 5 cmH_2O and FiO_2 0.4. Normal saline (5 mL/kg at 37-39 °C) was instilled into the tracheostomy tube, and then drained by gravity. Lavage was repeated until the partial pressure of oxygen in arterial blood (PaO_2) was lower than 120 mmHg at FiO_2 0.4 and PEEP 5 cmH_2O, for 30 min [S2]. Static compliance of respiratory system (C_{RS}) was measured by end-inspiratory and end-expiratory occlusions.

Study protocol

Before each test in normal and injured lung model, the animal was mechanically ventilated in the PS mode. Propofol and fentanyl were continuously infused and titrated to maintain no limbs movement but adequate and stable spontaneous breathing for at least 30 min. Then the animal was weaned from mechanical ventilation, and three tracheal oxygen treatments were performed in a randomly crossover fashion, lasting 20 min each:

1) Humidified T-piece oxygen

2) High-flow tracheal (HFT) oxygen via standard interface

3) HFT oxygen via modified interface

Humidified T-piece oxygen was delivered using an Oxyflo™ system composed of RT308 circuit and MR850 heated humidifier (Fisher & Paykel Healthcare, Auchland, New Zealand) at flow rate 10 L/min and temperature 37 °C. HFT was delivered via an AIRVO 2 device (Fisher & Paykel Healthcare, Auchland, New Zealand), and the manufacturer's standard assembly was equipped with a heated breathing circuit and an auto-fill humidification chamber (900PT501, Fisher & Paykel Healthcare, Auchland, New Zealand). HFT was set as flow rate 40 L/min, FiO_2 0.4 and temperature 37 °C.

Propofol and fentanyl were not adjusted during each sequence of tests. Tracheal oxygen treatment was terminated urgently and mechanical ventilation was initiated if the animal exhibited any of the following signs: agitation, heart rate (HR) above 130 beats/min or below 40 beats/min, MAP above 160 mmHg or below 65 mmHg, RR above 60 breaths/min or below 8 breaths/min, or pulse oxygen saturation below 88%.

References

S1. Baydur A, Behrakis PK, Zin WA, Jaeger M, Milic-Emili J. A simple method for assessing the validity of the esophageal balloon technique. Am Rev Respir Dis. 1982;126:788–91.

S2. Ranieri VM, Rubenfeld GD, Thompson BT, Ferguson ND, Caldwell E, Fan E, et al. Acute respiratory distress syndrome: the Berlin Definition. JAMA. 2012;307:2526–33.

Detailed methods of measurements

Pressure and flow derived parameters

Airway pressure (P_{aw}) and esophageal pressure (P_{es}) were measured by pressure transducers (KT 100D-2, Kleis TEK di CosimoMicelli, Italy, range: +/- 100 cmH_2O) connected to an ICU-Lab Pressure Box (ICU Lab, KleisTEK Engineering, Bari, Italy) by 80 cm rigid tube lines. Flow was measured by a heated Fleisch pneumotachograph (Vitalograph Inc, Lenexa, KS, USA) placed between the high-flow tracheal (HFT) oxygen interface and the tracheostomy tube. Pressure and flow signals were displayed continuously and saved (ICU-Lab 2.5 Software Package, ICU Lab, KleisTEK Engineering, Bari, Italy) in a laptop for further analysis, at a sample rate of 200 Hz.

At the end of each tested stage during T-piece oxygen, standard and modified high-flow oxygen therapy via tracheostomy (HFT), following data were collected and analyzed.

In the animal study, at the end of each tested phase (T-piece, standard or modified HFT), heart rate (HR) and mean arterial pressure (MAP), Partial pressure of end-tidal carbon dioxide ($P_{ET}CO_2$), partial pressure of carbon dioxide and oxygen in arterial blood ($PaCO_2$ and PaO_2) were collected.

The alveolar dead space fraction (V_D/V_T) was calculated as [S1]:

$$\text{Alveolar } V_D/V_T = \frac{PaCO_2 - P_{ET}CO_2}{PaCO_2}$$

Pressure and flow tracings in the last minute at each phase were analyzed, and the following parameters were collected as the average values of the last minutes:

1) The mean P_{aw} during either the inspiratory or expiratory phase [S2]

The mean P_{aw} during either the inspiratory or expiratory phase was measured. The points of zero flow rate were used to identify the onset inspiration and expiration.

2) The peak inspiratory and expiratory flow rate (PIF and PEF)

3) The inspiratory V_T, respiratory rate (RR) and minute ventilation (MV)

The inspiratory VT was integrated by flow tracing. RR was counted as the breaths in the last minute.

MV was the product of RR and VT.

4) The Pes swing during inspiration (ΔPes) [S3, S4].

ΔPes was used to assess the inspiratory effort.

5) The inspiratory and expiratory airway resistance [S5]

The inspiratory and expiratory airway resistance was estimated using the method introduced by Mead et al. Inspiratory and expiratory resistance was measured separately at flow rate of 200 mL/s. Change in Pes was used to calculate the resistance: $R = [(P_0\text{-}P)\text{-}(\frac{V}{C})]/\dot{V}$

where R is the resistance, P_0 is the Pes at the start of inspiratory or expiratory flow, V is the instantaneous volume integrated from flow, C is the dynamic compliance obtained for the same breath as the ratio of VT to ΔPes, and $\dot{V}$ is the instantaneous flow rate (= 0.2 L/s).

6) The intrinsic PEEP (PEEPi) [S3, S4] PEEPi was measured as the decrease in Pes immediately before the start of inspiratory flow.

7) The per-breath pressure time product (PTP) and the averaged PTP over a minute (PTPmin) [S6]

The per-breath PTP was derived by integrating the area of the Pes waveform during inspiration of each breath in the last minute. PTPmin was calculated as the sum of per breath PTP in the last minute.

8) The dynamic end-inspiratory and end-expiratory transpulmonary pressure (PL), and the driving transpulmonary pressure (ΔPL) [S3, S4]

The dynamic end-inspiratory and end-expiratory transpulmonary pressure (PL) were measured as the difference between Paw and the absolute Pes measured at the end of inspiration and end of expiration (all at zero flow). ΔP_L was calculated as the difference between end-inspiratory and end-expiratory P_L.

9) Inspired fraction of oxygen (FiO_2) [S7] and the PaO_2/FiO_2 ratio

FiO_2 was estimated as the sum of fresh gas volume and room air entrainment.

FiO_2 during T-piece oxygen was estimated as:

$$FiO_2 = \frac{(Ti\times167\times1.0) + (V_T - Ti\times167\times1.0)\times0.21}{V_T}$$

where Ti is inspiratory time (s), 167 represents T-piece oxygen flow rate (10 L/min = 167 mL/s), and 0.21 represents oxygen concentration in air.

Actual FiO_2 during HFT was estimated as:

$$FiO_2 = \frac{(Ti\times667\times0.40) + (V_T - Ti\times667\times0.40)\times0.21}{V_T}$$

where 667 represents HFT flow rate used in the present study (40 L/min = 667 mL/s) and 0.40 represents set HFT FiO_2.

Thus PaO_2/FiO_2 ratios in the three tracheal oxygen treatments were calculated.

Electrical impedance tomography (EIT) analysis

The tidal image of EIT is shown below. In off-line EIT analysis, we defined the thoracic cross-section using a matrix of 32×32 pixels. The dorsal 8×32 pixels of this matrix were discarded because no lung was contained in this region of the pig's anatomy [S8]. The remaining 24×32 pixels were defined as the global region of interest (ROI), which were further evenly divided into the ventral ROI (non-dependent lung region), middle ROI and dorsal ROI (dependent lung region) [S8, S9].

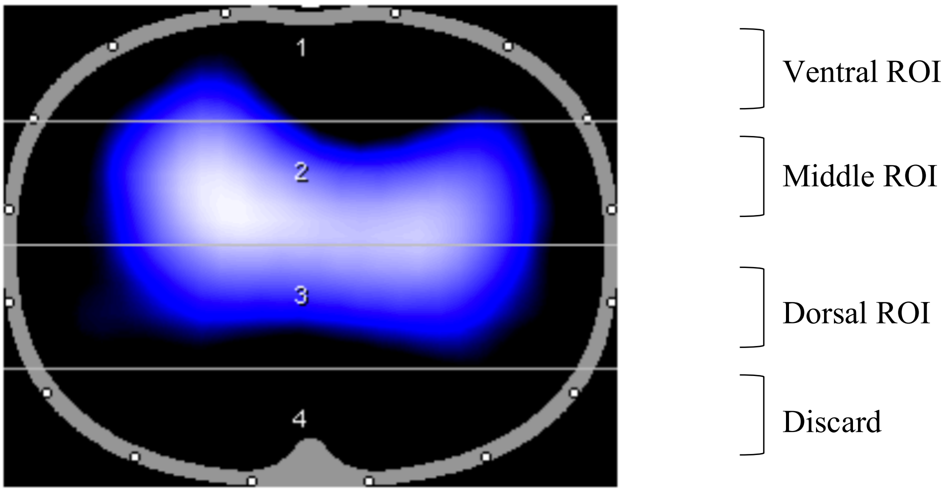

EIT measurements were collected in the last minute of each phase, including the following:

1) Considering T-piece as the baseline, global and regional changes in end-expiratory lung volume (ΔEELV) during HFT via the two interfaces were evaluated as the respective change in end-expiratory impedance multiplied by the ratio between V_T measured by flow integration (in mL) and the global tidal impedance change (in absolute unit) [S6, S10].

2) The regional distribution of tidal ventilation in the three ROIs was collected. The center of ventilation (COV) was calculated as the percentage of tidal ventilation distributed to the dorsal ROI in the global ROI [S11]. The higher the COV, the more tidal ventilation is distributed to the dependent lung region.

References

S1. Anderson CT, Breen PH. Carbon dioxide kinetics and capnography during critical care. Crit Care. 2000;4:207–15.

S2. Parke RL, McGuinness SP. Pressures delivered by nasal high flow oxygen during all phases of the respiratory cycle. Respir Care. 2013;58:1621–4.

S3. Akoumianaki E, Maggiore SM, Valenza F, Bellani G, Jubran A, Loring SH, et al. The application of esophageal pressure measurement in patients with respiratory failure. Am J Respir Crit Care Med. 2014;189:520–31.

S4. Mauri T, Yoshida T, Bellani G, Goligher EC, Carteaux G, Rittayamai N, et al. Esophageal and transpulmonary pressure in the clinical setting: meaning, usefulness and perspectives. Intensive Care Med. 2016;42:1360–73.

S5. Mead J, Whittenberger JL. Physical properties of human lungs measured during spontaneous respiration. J Appl Physiol. 1953; 5: 779–96.

S6. Mauri T, Turrini C, Eronia N, Grasselli G, Volta CA, Bellani G, et al. Physiologic Effects of High-Flow Nasal Cannula in Acute Hypoxemic Respiratory Failure. Am J Respir Crit Care Med. 2017;195:1207–15.

S7. Kacmarek RM, Stoller JK, Heuer AJ. Egan's fundamentals of respiratory care.11rd ed. American: Elsevier Health Sciences Division; 2016.

S8. Bodenstein M, Wang H, Boehme S, Vogt A, Kwiecien R, David M, et al. Influence of crystalloid and colloid fluid infusion and blood withdrawal on pulmonary bioimpedance in an animal model of mechanical ventilation. Physiol Meas. 2012;33:1225–36.

S9. Bodenstein M, Boehme S, Bierschock S, Vogt A, David M, et al. Determination of respiratory gas flow by electrical impedance tomography in an animal model of mechanical ventilation. BMC Pulm Med. 2014;14:73.

S10 Mauri T, Alban L, Turrini C, Cambiaghi B, Carlesso E, Taccone P, et al. Optimum support by high-flow nasal cannula in acute hypoxemic respiratory failure: effects of increasing flow rates. Intensive Care Med. 2017; 43:1453–63.

S11 Frerichs I, Dargaville PA, van Genderingen H, Morel DR, Rimensberger PC. Lung volume recruitment after surfactant administration modifies spatial distribution of ventilation. Am J Respir Crit Care Med. 2006;174:772–9.

Detailed bench experiment results

Condition 1

Compliance of breathing chamber=40 mL/cm H_2O

Inspiratory drive: normal (tidal volume of driving ventilator=300 mL)

Airway pressure (cm H_2O)

Entire respiratory cycle (mean)

		Flow (L/min)					
		10	20	30	40	50	60
Standard interface	mean	0.040	0.112	0.270	0.458	0.642	0.885
	SD	0.000	0.004	0.000	0.004	0.008	0.005
Modified interface	mean	0.208	0.762	1.577	2.780	4.345	6.168
	SD	0.008	0.017	0.008	0.013	0.005	0.039

Inspiratory (mean)

		Flow (L/min)					
		10	20	30	40	50	60
Standard interface	mean	-0.263	-0.205	-0.097	0.053	0.220	0.418
	SD	0.005	0.005	0.008	0.008	0.011	0.013
Modified interface	mean	-0.248	0.150	0.798	1.858	3.418	5.363
	SD	0.012	0.013	0.008	0.016	0.042	0.039

Inspiratory (minimal)

		Flow (L/min)					
		10	20	30	40	50	60
Standard interface	mean	-0.532	-0.488	-0.378	-0.273	-0.123	0.052
	SD	0.008	0.004	0.004	0.014	0.005	0.019
Modified interface	mean	-0.563	-0.280	0.305	1.288	2.888	4.887
	SD	0.005	0.011	0.008	0.013	0.010	0.049

Expiratory (mean)

		Flow (L/min)					
		10	20	30	40	50	60
Standard interface	mean	0.190	0.277	0.460	0.668	0.853	1.122
	SD	0.000	0.005	0.000	0.004	0.005	0.004
Modified interface	mean	0.435	1.080	1.973	3.247	4.752	6.455
	SD	0.008	0.015	0.010	0.012	0.008	0.036

Expiratory (maximal)

		Flow (L/min)					
		10	20	30	40	50	60
Standard interface	mean	1.540	1.713	1.930	2.173	2.420	2.693
	SD	0.013	0.018	0.017	0.005	0.006	0.015
Modified interface	mean	2.273	2.948	3.840	5.133	6.527	7.817
	SD	0.008	0.020	0.006	0.014	0.008	0.032

Pressure within the breathing chamber (cm H_2O)

Entire respiratory cycle (mean)

		Flow (L/min)					
		10	20	30	40	50	60
Standard interface	mean	0.130	0.200	0.355	0.542	0.728	0.972
	SD	0.000	0.000	0.005	0.004	0.008	0.004
Modified interface	mean	0.303	0.848	1.637	2.855	4.408	6.227
	SD	0.005	0.018	0.039	0.008	0.010	0.039

Inspiratory (mean)

		Flow (L/min)					
		10	20	30	40	50	60
Standard interface	mean	-0.302	-0.243	-0.140	0.010	0.198	0.378
	SD	0.004	0.008	0.006	0.009	0.004	0.010
Modified interface	mean	-0.278	0.115	0.757	1.822	3.402	5.347
	SD	0.008	0.015	0.008	0.018	0.021	0.043

Inspiratory (minimal)

		Flow (L/min)					
		10	20	30	40	50	60
Standard interface	mean	-1.032	-0.982	-0.815	-0.625	-0.462	-0.282
	SD	0.008	0.008	0.018	0.012	0.008	0.017
Modified interface	mean	-1.008	-0.607	-0.022	1.000	2.740	4.793
	SD	0.016	0.008	0.004	0.013	0.011	0.066

Expiratory (mean)

		Flow (L/min)					
		10	20	30	40	50	60
Standard interface	mean	0.348	0.430	0.607	0.818	1.008	1.273
	SD	0.004	0.006	0.005	0.004	0.008	0.005
Modified interface	mean	0.593	1.230	2.107	3.382	4.855	6.540
	SD	0.005	0.015	0.005	0.012	0.012	0.035

Expiratory (maximal)

		Flow (L/min)					
		10	20	30	40	50	60
Standard interface	mean	3.085	3.143	3.332	3.498	3.727	3.958
	SD	0.023	0.063	0.015	0.073	0.018	0.015
Modified interface	mean	3.618	4.192	4.918	6.120	7.320	8.463
	SD	0.019	0.023	0.013	0.014	0.013	0.027

Correlation of airway pressure with pressure within breathing chamber (pressure within breathing chamber = a*[airway pressure]+b)

	r	a	b
Entire respiratory cycle (mean)			
Standard interface	1.000	1.003	-0.088
Modified interface	1.000	1.005	-0.087
Inspiratory (mean)			
Standard interface	1.000	0.990	0.037
Modified interface	1.000	0.996	0.037
Inspiratory (minimal)			
Standard interface	0.995	0.750	0.234
Modified interface	1.000	0.945	0.335
Expiratory (mean)			
Standard interface	1.000	1.003	-0.155
Modified interface	1.000	1.012	-0.165
Expiratory (maximal)			
Standard interface	0.996	1.275	-2.331
Modified interface	1.000	1.140	-1.824

r = Pearson coefficient for correlation

Airway resistance (R_{aw})(cmH_2O/L/S)

Inspiratory Raw

		Flow (L/min)					
		10	20	30	40	50	60
Standard interface	mean	3.456	3.292	3.771	3.382	3.672	3.690
	SD	0.003	0.007	0.005	0.004	0.007	0.005
Modified interface	mean	4.188	4.517	5.096	6.050	5.467	6.542
	SD	0.005	0.005	0.005	0.011	0.012	0.035

Expiratory Raw

		Flow (L/min)					
		10	20	30	40	50	60
Standard interface	mean	5.134	5.470	5.500	5.676	5.776	5.552
	SD	0.023	0.043	0.015	0.003	0.018	0.015
Modified interface	mean	5.085	6.274	7.348	7.889	8.726	10.142
	SD	0.016	0.023	0.013	0.015	0.013	0.027

Condition 2

Compliance of breathing chamber=40 mL/cm H_2O

Inspiratory drive: normal (tidal volume of driving ventilator=600 mL)

Airway pressure (cm H_2O)

Entire respiratory cycle (mean)

		Flow (L/min)					
		10	20	30	40	50	60
Standard interface	mean	0.030	0.113	0.272	0.468	0.670	1.010
	SD	0.000	0.005	0.004	0.004	0.013	0.011
Modified interface	mean	0.290	0.860	1.632	2.881	4.392	6.148
	SD	0.011	0.017	0.008	0.063	0.029	0.021

Inspiratory (mean)

		Flow (L/min)					
		10	20	30	40	50	60
Standard interface	mean	-0.818	-0.792	-0.708	-0.582	-0.437	-0.213
	SD	0.008	0.010	0.008	0.012	0.014	0.010
Modified interface	mean	-0.920	-0.615	-0.168	0.780	2.253	3.970
	SD	0.006	0.015	0.019	0.035	0.056	0.042

Inspiratory (minimal)

		Flow (L/min)					
		10	20	30	40	50	60
Standard interface	mean	-1.548	-1.540	-1.472	-1.335	-1.243	-1.042
	SD	0.012	0.011	0.008	0.005	0.020	0.015
Modified interface	mean	-1.788	-1.517	-1.245	-0.475	0.978	2.777
	SD	0.010	0.015	0.018	0.022	0.026	0.023

Expiratory (mean)

		Flow (L/min)					
		10	20	30	40	50	60
Standard interface	mean	0.462	0.575	0.770	0.990	1.222	1.623
	SD	0.004	0.005	0.000	0.006	0.012	0.014
Modified interface	mean	0.898	1.598	2.537	3.928	5.425	7.138
	SD	0.015	0.026	0.005	0.035	0.034	0.139

Expiratory (maximal)

		Flow (L/min)					
		10	20	30	40	50	60
Standard interface	mean	2.737	2.917	3.167	3.463	3.752	4.142
	SD	0.015	0.019	0.018	0.032	0.016	0.018
Modified interface	mean	4.072	4.623	5.663	7.142	8.570	10.150
	SD	0.015	0.051	0.029	0.043	0.105	0.026

Pressure within the breathing chamber (cm H_2O)

Entire respiratory cycle (mean)

		Flow (L/min)					
		10	20	30	40	50	60
Standard interface	mean	0.217	0.292	0.450	0.633	0.832	1.157
	SD	0.005	0.004	0.000	0.005	0.021	0.043
Modified interface	mean	0.450	1.005	1.763	2.980	4.500	6.242
	SD	0.011	0.022	0.008	0.034	0.028	0.020

Inspiratory (mean)

		Flow (L/min)					
		10	20	30	40	50	60
Standard interface	mean	-1.045	-1.108	-0.938	-0.808	-0.662	-0.435
	SD	0.010	0.013	0.008	0.019	0.017	0.015
Modified interface	mean	-1.163	-0.868	-0.403	0.545	2.067	3.788
	SD	0.005	0.017	0.015	0.038	0.056	0.043

Inspiratory (minimal)

		Flow (L/min)					
		10	20	30	40	50	60
Standard interface	mean	-2.300	-2.282	-2.195	-2.092	-1.942	-1.725
	SD	0.013	0.015	0.005	0.018	0.019	0.035
Modified interface	mean	-2.407	-2.192	-1.848	-1.028	0.545	2.415
	SD	0.114	0.013	0.024	0.044	0.037	0.030

Expiratory (mean)

		Flow (L/min)					
		10	20	30	40	50	60
Standard interface	mean	0.853	0.945	1.152	1.348	1.582	1.980
	SD	0.010	0.042	0.004	0.013	0.012	0.014
Modified interface	mean	1.263	1.942	2.852	4.228	5.682	7.318
	SD	0.005	0.026	0.004	0.036	0.035	0.060

Expiratory (maximal)

		Flow (L/min)					
		10	20	30	40	50	60
Standard interface	mean	5.460	5.570	5.745	5.932	6.152	6.488
	SD	0.030	0.017	0.015	0.031	0.027	0.029
Modified interface	mean	6.302	6.813	7.552	8.827	10.063	11.413
	SD	0.018	0.037	0.028	0.046	0.034	0.020

Correlation of airway pressure with pressure within breathing chamber (pressure within breathing chamber = a*[airway pressure]+b)

	r	a	b
Entire respiratory cycle (mean)			
Standard interface	1.000	1.040	-0.193
Modified interface	1.000	1.011	-0.154
Inspiratory (mean)			
Standard interface	0.992	0.915	0.170
Modified interface	1.000	0.985	0.232
Inspiratory (minimal)			
Standard interface	0.997	0.887	0.490
Modified interface	1.000	0.938	0.494
Expiratory (mean)			
Standard interface	1.000	1.027	-0.405
Modified interface	1.000	1.028	-0.403
Expiratory (maximal)			
Standard interface	0.998	1.375	-4.735
Modified interface	1.000	1.192	-3.423

r = Pearson coefficient for correlation

Airway resistance (R_{aw})($cmH_2O/L/S$)

Inspiratory Raw

		Flow (L/min)					
		10	20	30	40	50	60
Standard interface	mean	4.007	3.930	3.915	4.253	4.266	4.746
	SD	0.003	0.008	0.008	0.005	0.007	0.005
Modified interface	mean	4.580	4.548	5.733	6.664	6.848	3.153
	SD	0.005	0.005	0.005	0.007	0.010	0.004

Expiratory Raw

		Flow (L/min)					
		10	20	30	40	50	60
Standard interface	mean	6.575	6.619	6.862	6.863	6.854	6.948
	SD	0.013	0.033	0.025	0.003	0.018	0.015
Modified interface	mean	8.260	8.592	8.793	9.752	9.993	10.859
	SD	0.016	0.023	0.013	0.016	0.013	0.023

Condition 3
Compliance of breathing chamber=40 mL/cm H_2O
Inspiratory drive: normal (tidal volume of driving ventilator=900 mL)

Airway pressure (cm H_2O)

Entire respiratory cycle (mean)

		Flow (L/min)					
		10	20	30	40	50	60
Standard interface	mean	-0.023	0.058	0.205	0.360	0.592	0.808
	SD	0.005	0.004	0.005	0.000	0.004	0.004
Modified interface	mean	0.317	1.042	1.748	2.812	4.287	6.117
	SD	0.016	0.035	0.034	0.044	0.041	0.059

Inspiratory (mean)

		Flow (L/min)					
		10	20	30	40	50	60
Standard interface	mean	-1.672	-1.640	-1.570	-1.508	-1.390	-1.237
	SD	0.019	0.024	0.023	0.012	0.011	0.027
Modified interface	mean	-1.952	-1.538	-1.162	-0.532	0.725	2.383
	SD	0.027	0.022	0.032	0.052	0.064	0.084

Inspiratory (minimal)

		Flow (L/min)					
		10	20	30	40	50	60
Standard interface	mean	-3.065	-3.053	-2.980	-2.967	-2.865	-2.723
	SD	0.027	0.005	0.014	0.024	0.023	0.015
Modified interface	mean	-3.643	-3.185	-2.905	-2.468	-1.352	0.377
	SD	0.039	0.078	0.026	0.025	0.044	0.077

Expiratory (mean)

		Flow (L/min)					
		10	20	30	40	50	60
Standard interface	mean	0.795	0.917	1.100	1.292	1.585	1.842
	SD	0.005	0.008	0.006	0.004	0.005	0.008
Modified interface	mean	1.463	2.328	3.248	4.543	6.090	7.868
	SD	0.016	0.084	0.035	0.048	0.037	0.053

Expiratory (maximal)

		Flow (L/min)					
		10	20	30	40	50	60
Standard interface	mean	3.745	3.898	4.165	4.403	4.752	4.998
	SD	0.022	0.015	0.021	0.025	0.016	0.013
Modified interface	mean	5.747	6.148	7.213	8.668	10.340	12.153
	SD	0.015	0.110	0.080	0.064	0.063	0.075

Pressure within the breathing chamber (cm H_2O)

Entire respiratory cycle (mean)

		Flow (L/min)					
		10	20	30	40	50	60
Standard interface	mean	0.272	0.355	0.490	0.645	0.867	1.083
	SD	0.004	0.005	0.000	0.005	0.008	0.005
Modified interface	mean	0.565	1.298	1.957	3.003	4.462	6.260
	SD	0.019	0.035	0.034	0.042	0.041	0.056

Inspiratory (mean)

		Flow (L/min)					
		10	20	30	40	50	60
Standard interface	mean	-2.192	-2.143	-2.088	-2.007	-1.887	-1.720
	SD	0.028	0.031	0.029	0.019	0.018	0.031
Modified interface	mean	-2.472	-2.050	-1.707	-1.073	0.265	1.892
	SD	0.033	0.030	0.038	0.057	0.067	0.064

Inspiratory (minimal)

		Flow (L/min)					
		10	20	30	40	50	60
Standard interface	mean	-4.212	-4.127	-4.090	-4.032	-3.910	-3.757
	SD	0.040	0.019	0.028	0.025	0.022	0.034
Modified interface	mean	-4.742	-4.307	-4.045	-3.590	-2.250	-0.510
	SD	0.055	0.054	0.059	0.028	0.051	0.068

Expiratory (mean)

		Flow (L/min)					
		10	20	30	40	50	60
Standard interface	mean	1.558	1.617	1.788	1.970	2.253	2.505
	SD	0.138	0.014	0.008	0.006	0.010	0.014
Modified interface	mean	2.102	2.953	3.800	5.120	6.598	8.302
	SD	0.015	0.079	0.104	0.046	0.039	0.056

Expiratory (maximal)

		Flow (L/min)					
		10	20	30	40	50	60
Standard interface	mean	7.657	7.753	7.917	8.115	8.355	8.523
	SD	0.028	0.026	0.029	0.046	0.037	0.043
Modified interface	mean	9.013	9.507	10.260	11.535	12.885	14.420
	SD	0.019	0.099	0.108	0.073	0.064	0.060

Correlation of airway pressure with pressure within breathing chamber (pressure within breathing chamber = a*[airway pressure]+b)

	r	a	b
Entire respiratory cycle (mean)			
Standard interface	1.000	1.028	-0.303
Modified interface	1.000	1.020	-0.261
Inspiratory (mean)			
Standard interface	0.999	0.931	0.364
Modified interface	1.000	0.988	0.502
Inspiratory (minimal)			
Standard interface	0.986	0.776	0.178
Modified interface	0.999	0.933	0.828
Expiratory (mean)			
Standard interface	0.998	1.077	-0.844
Modified interface	1.000	1.031	-0.706
Expiratory (maximal)			
Standard interface	0.999	1.427	-7.163
Modified interface	1.000	1.200	-5.144

r = Pearson coefficient for correlation

Airway resistance (R_{aw})($cmH_2O/L/S$)

Inspiratory Raw

		Flow (L/min)					
		10	20	30	40	50	60
Standard interface	mean	4.247	4.286	4.681	4.743	4.635	4.728
	SD	0.003	0.009	0.008	0.015	0.007	0.005
Modified interface	mean	4.487	5.082	6.382	6.370	6.842	4.932
	SD	0.009	0.006	0.005	0.019	0.010	0.004

Expiratory Raw

		Flow (L/min)					
		10	20	30	40	50	60
Standard interface	mean	7.603	7.443	7.197	7.592	7.856	7.322
	SD	0.016	0.003	0.025	0.003	0.018	0.015
Modified interface	mean	9.743	9.322	10.145	10.555	11.782	12.408
	SD	0.016	0.023	0.013	0.016	0.013	0.023

Condition 4

Compliance of breathing chamber=60 mL/cm H_2O

Inspiratory drive: normal (tidal volume of driving ventilator=300 mL)

Airway pressure (cm H_2O)

Entire respiratory cycle (mean)

		Flow (L/min)					
		10	20	30	40	50	60
Standard interface	mean	0.070	0.148	0.272	0.443	0.702	0.978
	SD	0.000	0.004	0.004	0.010	0.021	0.004
Modified interface	mean	0.118	0.653	1.523	2.748	4.462	6.077
	SD	0.010	0.015	0.015	0.027	0.012	0.043

Inspiratory (mean)

		Flow (L/min)					
		10	20	30	40	50	60
Standard interface	mean	-0.252	-0.210	-0.117	0.023	0.277	0.543
	SD	0.004	0.006	0.005	0.019	0.060	0.018
Modified interface	mean	-0.352	0.007	0.720	1.892	3.748	5.723
	SD	0.022	0.010	0.023	0.028	0.030	0.073

Inspiratory (minimal)

		Flow (L/min)					
		10	20	30	40	50	60
Standard interface	mean	-0.528	-0.498	-0.420	-0.247	-0.077	0.175
	SD	0.008	0.004	0.006	0.108	0.054	0.008
Modified interface	mean	-0.717	-0.368	0.180	1.315	3.280	5.510
	SD	0.030	0.161	0.020	0.031	0.011	0.047

Expiratory (mean)

		Flow (L/min)					
		10	20	30	40	50	60
Standard interface	mean	0.238	0.330	0.475	0.662	0.930	1.210
	SD	0.004	0.000	0.005	0.004	0.009	0.014
Modified interface	mean	0.375	0.992	1.963	3.178	4.725	6.153
	SD	0.012	0.015	0.015	0.027	0.023	0.024

Expiratory (maximal)

		Flow (L/min)					
		10	20	30	40	50	60
Standard interface	mean	1.413	1.553	1.698	1.885	2.173	2.468
	SD	0.005	0.015	0.004	0.008	0.014	0.004
Modified interface	mean	1.880	2.545	3.493	4.667	5.735	6.400
	SD	0.021	0.020	0.025	0.030	0.008	0.314

Pressure within the breathing chamber (cm H_2O)

Entire respiratory cycle (mean)

		Flow (L/min)					
		10	20	30	40	50	60
Standard interface	mean	0.130	0.203	0.330	0.502	0.760	1.038
	SD	0.000	0.005	0.000	0.008	0.021	0.008
Modified interface	mean	0.170	0.698	1.563	2.783	4.480	6.102
	SD	0.011	0.015	0.015	0.027	0.013	0.046

Inspiratory (mean)

		Flow (L/min)					
		10	20	30	40	50	60
Standard interface	mean	-0.328	-0.292	-0.195	-0.055	0.205	0.472
	SD	0.008	0.010	0.005	0.019	0.063	0.018
Modified interface	mean	-0.433	-0.080	0.637	1.823	3.700	5.708
	SD	0.023	0.014	0.024	0.027	0.030	0.077

Inspiratory (minimal)

		Flow (L/min)					
		10	20	30	40	50	60
Standard interface	mean	-0.995	-0.978	-0.855	-0.717	-0.455	-0.337
	SD	0.012	0.004	0.014	0.024	0.102	0.008
Modified interface	mean	-1.158	-0.862	-0.220	1.100	3.170	5.285
	SD	0.032	0.012	0.021	0.036	0.011	0.042

Expiratory (mean)

		Flow (L/min)					
		10	20	30	40	50	60
Standard interface	mean	0.370	0.463	0.600	0.792	1.058	1.333
	SD	0.000	0.005	0.006	0.004	0.008	0.010
Modified interface	mean	0.495	1.107	2.062	3.263	4.777	6.188
	SD	0.015	0.015	0.017	0.023	0.027	0.026

Expiratory (maximal)

		Flow (L/min)					
		10	20	30	40	50	60
Standard interface	mean	2.722	2.807	2.920	3.077	3.303	3.553
	SD	0.004	0.043	0.009	0.005	0.022	0.016
Modified interface	mean	3.050	3.593	4.375	5.403	6.235	6.938
	SD	0.019	0.020	0.031	0.029	0.008	0.031

Correlation of airway pressure with pressure within breathing chamber (pressure within breathing chamber = a*[airway pressure]+b)

	r	a	b
Entire respiratory cycle (mean)			
Standard interface	1.000	0.998	-0.057
Modified interface	1.000	1.005	-0.049
Inspiratory (mean)			
Standard interface	1.000	0.989	0.076
Modified interface	1.000	0.989	0.085
Inspiratory (minimal)			
Standard interface	0.986	0.985	0.446
Modified interface	0.999	0.952	0.372
Expiratory (mean)			
Standard interface	1.000	1.007	-0.134
Modified interface	1.000	1.016	-0.131
Expiratory (maximal)			
Standard interface	0.999	1.254	-1.976
Modified interface	1.000	1.174	-1.672

r = Pearson coefficient for correlation

Airway resistance (R_{aw})($cmH_2O/L/S$)

Inspiratory Raw

		Flow (L/min)					
		10	20	30	40	50	60
Standard interface	mean	3.217	2.994	2.682	3.425	3.394	3.578
	SD	0.009	0.006	0.005	0.019	0.010	0.004
Modified interface	mean	4.518	3.697	5.500	6.150	4.734	4.303
	SD	0.016	0.003	0.025	0.003	0.018	0.015

Expiratory Raw

		Flow (L/min)					
		10	20	30	40	50	60
Standard interface	mean	4.887	5.132	5.533	5.192	5.597	5.647
	SD	0.016	0.023	0.013	0.016	0.018	0.015
Modified interface	mean	6.971	7.055	7.606	10.121	11.125	13.067
	SD	0.016	0.033	0.031	0.016	0.013	0.023

Condition 5
Compliance of breathing chamber=60 mL/cm H_2O
Inspiratory drive: normal (tidal volume of driving ventilator=600 mL)

Airway pressure (cm H_2O)

Entire respiratory cycle (mean)

		Flow (L/min)					
		10	20	30	40	50	60
Standard interface	mean	0.370	0.463	0.600	0.792	1.058	1.333
	SD	0.000	0.005	0.006	0.004	0.008	0.010
Modified interface	mean	0.495	1.107	2.062	3.263	4.777	6.188
	SD	0.015	0.015	0.017	0.023	0.027	0.026

Inspiratory (mean)

		Flow (L/min)					
		10	20	30	40	50	60
Standard interface	mean	2.722	2.807	2.920	3.077	3.303	3.553
	SD	0.004	0.043	0.009	0.005	0.022	0.016
Modified interface	mean	3.050	3.593	4.375	5.403	6.235	6.938
	SD	0.019	0.020	0.031	0.029	0.008	0.031

Inspiratory (minimal)

		Flow (L/min)					
		10	20	30	40	50	60
Standard interface	mean	0.042	0.130	0.267	0.502	0.692	0.955
	SD	0.004	0.011	0.014	0.008	0.012	0.023
Modified interface	mean	0.073	0.698	1.530	2.790	4.472	6.068
	SD	0.012	0.015	0.009	0.045	0.010	0.052

Expiratory (mean)

		Flow (L/min)					
		10	20	30	40	50	60
Standard interface	mean	0.475	0.567	0.715	0.907	1.263	1.557
	SD	0.005	0.005	0.005	0.008	0.010	0.008
Modified interface	mean	0.877	1.548	2.568	3.642	5.047	6.580
	SD	0.010	0.033	0.031	0.017	0.015	0.025

Expiratory (maximal)

		Flow (L/min)					
		10	20	30	40	50	60
Standard interface	mean	2.378	2.510	2.702	2.927	3.317	3.597
	SD	0.008	0.000	0.015	0.012	0.008	0.012
Modified interface	mean	3.388	4.007	5.078	6.163	7.487	8.500
	SD	0.015	0.040	0.029	0.019	0.008	0.025

Pressure within the breathing chamber (cm H_2O)

Entire respiratory cycle (mean)

		Flow (L/min)					
		10	20	30	40	50	60
Standard interface	mean	0.142	0.207	0.322	0.483	0.765	1.028
	SD	0.004	0.005	0.004	0.005	0.008	0.004
Modified interface	mean	0.347	0.875	1.685	2.695	4.170	5.953
	SD	0.008	0.024	0.027	0.021	0.006	0.021

Inspiratory (mean)

		Flow (L/min)					
		10	20	30	40	50	60
Standard interface	mean	-1.192	-1.175	-1.110	-0.982	-0.830	-0.642
	SD	0.030	0.015	0.011	0.026	0.023	0.017
Modified interface	mean	-1.350	-1.048	-0.563	0.307	1.850	3.832
	SD	0.014	0.026	0.010	0.019	0.024	0.070

Inspiratory (minimal)

		Flow (L/min)					
		10	20	30	40	50	60
Standard interface	mean	-2.492	-2.455	-2.403	-2.310	-2.150	-1.958
	SD	0.022	0.019	0.022	0.028	0.022	0.019
Modified interface	mean	-2.722	-2.417	-2.060	-1.197	0.462	2.673
	SD	0.034	0.040	0.026	0.028	0.019	0.042

Expiratory (mean)

		Flow (L/min)					
		10	20	30	40	50	60
Standard interface	mean	0.837	0.917	1.058	1.240	1.590	1.880
	SD	0.008	0.005	0.004	0.017	0.019	0.013
Modified interface	mean	1.205	1.858	2.848	3.890	5.227	6.700
	SD	0.015	0.033	0.034	0.024	0.014	0.025

Expiratory (maximal)

		Flow (L/min)					
		10	20	30	40	50	60
Standard interface	mean	4.602	4.655	4.770	4.953	5.245	5.445
	SD	0.029	0.020	0.024	0.015	0.018	0.024
Modified interface	mean	5.283	5.797	6.632	7.513	8.658	9.458
	SD	0.031	0.034	0.027	0.035	0.017	0.019

Correlation of airway pressure with pressure within breathing chamber (pressure within breathing chamber = a*[airway pressure]+b)

	r	a	b
Entire respiratory cycle (mean)			
Standard interface	0.999	1.070	0.244
Modified interface	0.997	1.030	0.284
Inspiratory (mean)			
Standard interface	0.996	1.449	4.496
Modified interface	0.954	0.728	4.565
Inspiratory (minimal)			
Standard interface	0.987	1.689	4.306
Modified interface	0.981	1.090	3.560
Expiratory (mean)			
Standard interface	1.000	1.034	-0.382
Modified interface	1.000	1.038	-0.383
Expiratory (maximal)			
Standard interface	0.998	1.398	-4.007
Modified interface	1.000	1.221	-3.050

r = Pearson coefficient for correlation

Airway resistance (R_{aw})($cmH_2O/L/S$)

Inspiratory Raw

		Flow (L/min)					
		10	20	30	40	50	60
Standard interface	mean	4.053	3.656	3.913	4.268	4.222	4.935
	SD	0.019	0.004	0.015	0.031	0.010	0.004
Modified interface	mean	4.210	4.079	4.836	6.110	6.523	6.823
	SD	0.016	0.005	0.025	0.013	0.018	0.015

Expiratory Raw

		Flow (L/min)					
		10	20	30	40	50	60
Standard interface	mean	6.068	5.960	6.278	6.334	6.579	6.848
	SD	0.012	0.023	0.013	0.016	0.018	0.015
Modified interface	mean	7.696	7.415	8.675	8.884	10.425	11.498
	SD	0.016	0.023	0.031	0.016	0.013	0.023

Condition 6

Compliance of breathing chamber=60 mL/cm H_2O

Inspiratory drive: normal (tidal volume of driving ventilator=900 mL)

Airway pressure (cm H_2O)

Entire respiratory cycle (mean)

		Flow (L/min)					
		10	20	30	40	50	60
Standard interface	mean	-0.103	-0.002	0.150	0.310	0.500	0.760
	SD	0.005	0.004	0.000	0.000	0.000	0.006
Modified interface	mean	0.150	0.872	1.657	2.666	4.178	6.155
	SD	0.017	0.056	0.021	0.153	0.012	0.016

Inspiratory (mean)

		Flow (L/min)					
		10	20	30	40	50	60
Standard interface	mean	-1.870	-1.842	-1.743	-1.700	-1.605	-1.445
	SD	0.014	0.027	0.018	0.022	0.023	0.023
Modified interface	mean	-2.275	-1.858	-1.507	-0.753	0.608	2.552
	SD	0.019	0.041	0.005	0.070	0.059	0.021

Inspiratory (minimal)

		Flow (L/min)					
		10	20	30	40	50	60
Standard interface	mean	-3.397	-3.353	-3.283	-3.273	-3.180	-3.033
	SD	0.016	0.030	0.012	0.021	0.011	0.008
Modified interface	mean	-4.067	-3.588	-3.293	-2.640	-1.248	0.780
	SD	0.031	0.035	0.029	0.045	0.022	0.015

Expiratory (mean)

		Flow (L/min)					
		10	20	30	40	50	60
Standard interface	mean	0.790	0.933	1.122	1.432	1.565	1.890
	SD	0.011	0.018	0.008	0.249	0.008	0.020
Modified interface	mean	1.375	2.268	3.268	4.448	5.818	7.577
	SD	0.018	0.083	0.021	0.029	0.019	0.015

Expiratory (maximal)

		Flow (L/min)					
		10	20	30	40	50	60
Standard interface	mean	3.253	3.412	3.655	3.923	4.197	4.563
	SD	0.015	0.013	0.010	0.020	0.015	0.014
Modified interface	mean	4.720	5.347	6.507	7.757	9.005	10.598
	SD	0.011	0.059	0.059	0.048	0.010	0.025

Pressure within the breathing chamber (cm H_2O)

Entire respiratory cycle (mean)

		Flow (L/min)					
		10	20	30	40	50	60
Standard interface	mean	0.115	0.247	0.390	0.540	0.712	0.980
	SD	0.054	0.005	0.000	0.000	0.004	0.011
Modified interface	mean	0.382	1.085	1.848	2.885	4.307	6.242
	SD	0.016	0.055	0.024	0.046	0.014	0.010

Inspiratory (mean)

		Flow (L/min)					
		10	20	30	40	50	60
Standard interface	mean	-2.440	-2.417	-2.317	-2.283	-2.195	-2.008
	SD	0.036	0.010	0.021	0.027	0.031	0.026
Modified interface	mean	-2.815	-2.418	-1.993	-1.205	0.210	2.195
	SD	0.042	0.032	0.008	0.057	0.015	0.045

Inspiratory (minimal)

		Flow (L/min)					
		10	20	30	40	50	60
Standard interface	mean	-4.568	-4.613	-4.480	-4.472	-4.405	-4.217
	SD	0.060	0.029	0.040	0.071	0.028	0.039
Modified interface	mean	-5.162	-4.673	-4.355	-3.623	-2.148	0.158
	SD	0.035	0.039	0.029	0.046	0.046	0.013

Expiratory (mean)

		Flow (L/min)					
		10	20	30	40	50	60
Standard interface	mean	1.437	1.600	1.773	1.975	2.190	2.505
	SD	0.046	0.020	0.010	0.015	0.013	0.025
Modified interface	mean	1.998	2.862	3.817	4.953	6.230	7.828
	SD	0.015	0.084	0.030	0.028	0.021	0.013

Expiratory (maximal)

		Flow (L/min)					
		10	20	30	40	50	60
Standard interface	mean	6.468	6.687	6.832	7.005	7.227	7.447
	SD	0.078	0.034	0.037	0.045	0.014	0.037
Modified interface	mean	7.615	8.152	9.010	10.062	11.062	12.165
	SD	0.020	0.059	0.083	0.047	0.052	0.026

Correlation of airway pressure with pressure within breathing chamber (pressure within breathing chamber = a*[airway pressure]+b)

	r	a	b
Entire respiratory cycle (mean)			
Standard interface	0.999	1.020	-0.238
Modified interface	1.000	1.025	-0.247
Inspiratory (mean)			
Standard interface	0.998	0.991	0.556
Modified interface	1.000	0.960	0.426
Inspiratory (minimal)			
Standard interface	0.974	0.911	0.808
Modified interface	1.000	0.911	0.662
Expiratory (mean)			
Standard interface	0.995	1.049	-0.719
Modified interface	1.000	1.062	-0.775
Expiratory (maximal)			
Standard interface	0.995	1.371	-5.689
Modified interface	1.000	1.281	-5.074

r = Pearson coefficient for correlation

Airway resistance (R_{aw})($cmH_2O/L/S$)

Inspiratory Raw

		Flow (L/min)					
		10	20	30	40	50	60
Standard interface	mean	4.119	4.425	4.381	4.585	4.677	4.808
	SD	0.011	0.014	0.021	0.031	0.010	0.014
Modified interface	mean	4.949	5.467	5.712	6.283	6.997	7.425
	SD	0.012	0.013	0.025	0.013	0.018	0.015

Expiratory Raw

		Flow (L/min)					
		10	20	30	40	50	60
Standard interface	mean	6.741	6.652	6.990	7.221	7.220	7.293
	SD	0.031	0.023	0.023	0.016	0.018	0.015
Modified interface	mean	7.712	7.196	9.852	10.665	11.136	13.457
	SD	0.010	0.023	0.031	0.013	0.013	0.023

Figure S1. Relationship between airway pressure and flow rate with modified high-flow tracheal oxygen

Additional expiratory resistance elevates airway pressure and lung volume during high-flow tracheal oxygen via tracheostomy

Guang-Qiang Chen, Xiu-Mei Sun, Yu-Mei Wang, Yi-Min Zhou, Jing-Ran Chen, Kun-Ming Cheng, Yan-Lin Yang, Ming Xu, Jian-Xin Zhou

Additional file 5, Figure S1. Relationship between the airway pressure and flow rate with modified high-flow tracheal oxygen

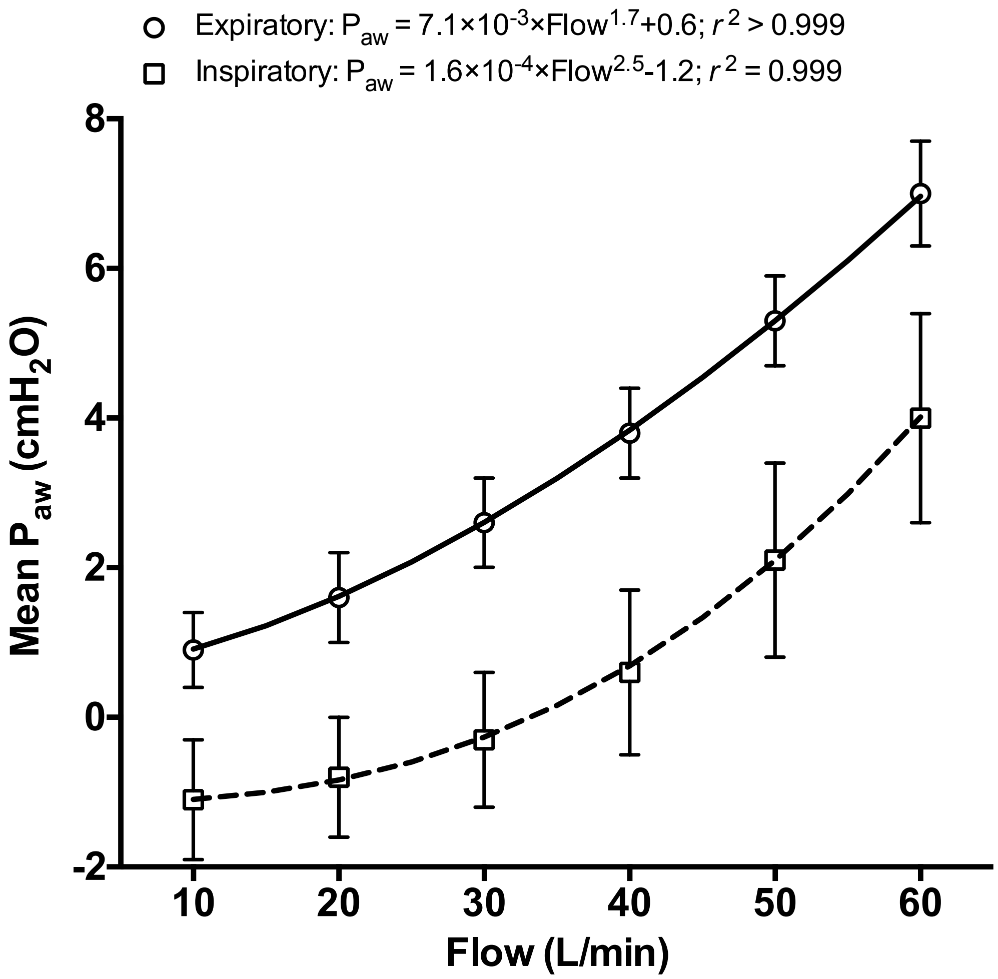

Figure S1 shows the relationship of the airway pressure (P_{aw}) and flow rate with modified high-flow tracheal (HFT) oxygen therapy via tracheostomy in the bench experiment. The P_{aw} and flow rate were fitted by a power equation:

$$P_{aw} = a \times Flow^{b} + c$$

The coefficient of determination (r^2) is also shown.

Table S1. Multiple linear regression for determinants of mean expiratory airway pressure during modified high-flow tracheal oxygen

Table S1. Multiple linear regression for determinants of mean expiratory airway pressure during modified high-flow tracheal oxygen

Determinants	Coefficient (95% confidence interval)	Standardized coefficient	p
Flow^2	0.001 (0.001, 0.002)	0.755	<0.001
Expiratory resistance	0.30 (0.17, 0.43)	0.265	<0.001

r^2, coefficient of determination.

Table S2. Characteristics before and after the induction of lung injury

Tables S2. Characteristics before and after the induction of lung injury (n = 6)

Variables	Before	After	*p*
PaO_2/FiO_2	352 ± 77	228 ± 45	0.003
$PaCO_2$, mm Hg	50 ± 5	56 ± 3	0.011
$P_{ET}CO_2$, mm Hg	39 ± 4	38 ± 5	0.380
Alveolar dead space fraction	0.22 ± 0.10	0.33 ± 0.05	0.019
C_{RS}, mL/cmH_2O	40 ± 5	26 ± 1	< 0.001
HR, beat/min	83 ± 7	81 ± 7	0.582
MAP, mm Hg	119 ± 14	121 ± 15	0.846

C_{RS}, respiratory system compliance; FiO_2, fraction of inspired oxygen; HR, heart rate; MAP, mean arterial pressure; PaO_2, partial pressure of oxygen in arterial blood; $PaCO_2$, partial pressure of carbon dioxide in arterial blood.

Data are shown as mean ± standard deviation.
